# Computational Model of MicroRNA Control of HIF-VEGF Pathway: Insights into the Pathophysiology of Ischemic Vascular Disease and Cancer

**DOI:** 10.1371/journal.pcbi.1004612

**Published:** 2015-11-20

**Authors:** Chen Zhao, Aleksander S. Popel

**Affiliations:** Department of Biomedical Engineering, School of Medicine, Johns Hopkins University, Baltimore, Maryland, United States of America; University of Virginia, UNITED STATES

## Abstract

HRMs (hypoxia-responsive miRNAs) are a specific group of microRNAs that are regulated by hypoxia. Recent studies revealed that several HRMs including let-7 family miRNAs were highly induced in response to HIF (hypoxia-inducible factor) stabilization in hypoxia, and they potently participated in angiogenesis by targeting AGO1 (argonaute 1) and upregulating VEGF (vascular endothelial growth factor). Here we constructed a novel computational model of microRNA control of HIF-VEGF pathway in endothelial cells to quantitatively investigate the role of HRMs in modulating the cellular adaptation to hypoxia. The model parameters were optimized and the simulations based on these parameters were validated against several published *in vitro* experimental data. To advance the mechanistic understanding of oxygen sensing in hypoxia, we demonstrated that the rate of HIF-1α nuclear import substantially influences its stabilization and the formation of HIF-1 transcription factor complex. We described the biological feedback loops involving let-7 and AGO1 in which the impact of external perturbations were minimized; as a pair of master regulators when low oxygen tension was sensed, they coordinated the critical process of VEGF desuppression in a controlled manner. Prompted by the model-motivated discoveries, we proposed and assessed novel pathway-specific therapeutics that modulate angiogenesis by adjusting VEGF synthesis in tumor and ischemic cardiovascular disease. Through simulations that capture the complex interactions between miRNAs and miRNA-processing molecules, this model explores an innovative perspective about the distinctive yet integrated roles of different miRNAs in angiogenesis, and it will help future research to elucidate the dysregulated miRNA profiles found in cancer and various cardiovascular diseases.

## Author Summary

Cells living in a hypoxic environment secrete signals to stimulate new blood vessel growth, a process termed angiogenesis, to acquire more oxygen and nutrients. Hypoxia-inducible factor 1 (HIF-1) accumulates in hypoxia and expedites the release of pro-angiogenic cytokines such as vascular endothelial growth factor (VEGF), a prime inducer of angiogenesis. The intermediate signaling events connecting HIF-1 and VEGF are tightly controlled by microRNAs (miRs), which are endogenous, non-coding RNA molecules and powerful regulators in cancer and cardiovascular disease. Given the importance of angiogenesis in tumor development and post-ischemia reperfusion, it holds great basic research and therapeutic value to investigate how miRs modulate intracellular VEGF synthesis to control angiogenesis in hypoxia. We present a computational model that details the interactions between miRs and other key molecules which make up different hierarchies in HIF-miR-VEGF pathway. Based on simulation analysis, new potential therapies are introduced and tested *in silico*, from which the strategies that most effectively reduce VEGF synthesis in cancer, or enhance VEGF release in ischemic vascular disease are identified. We conclude that in hypoxia different miRs work consonantly to fine-tune the cellular adaptations; when a master miR alters its expression, dynamics of other miRs vary accordingly which together contribute to aberrant RNA/protein profiles observed in the pathophysiology of multiple diseases.

## Introduction

When cells are exposed to low oxygen tension, cellular adaptation occurs by transcriptionally activating a variety of genes that participate in pathways involving angiogenesis, metabolism and proliferation/survival [1]. The oxygen-sensitive transcription factor HIF-1 (hypoxia-inducible factor 1), with over 1000 putative targets in human, is the master mediator of this response [2, 3]. HIF-1 is a heterodimer of HIF-1β subunit, which is constitutively expressed regardless of O_2_ availability, and HIF-1α subunit, whose expression is highly dependent on O_2_ levels. In normoxia, HIF-1α protein levels are undetectable [4]; they are rapidly hydroxylated by PHDs (prolyl hydroxylases) and FIH-1 (factor inhibiting HIF-1, abbreviated throughout this paper as FIH), followed by polyubiquitination by pVHL (von Hippel-Lindau ubiquitin E3 ligase) complex that marks HIF-1α for proteasomal degradation [1, 5]. In hypoxic conditions, HIF-1α protein is stabilized and it translocates from the cytoplasm into the nucleus, where it binds to HIF-1β to form the heterodimer HIF-1 complex. The dimer complex associates with HREs (hypoxia-responsive elements) located in the promoters of target genes and tethers transcriptional coactivators, such as CBP (CREB-binding protein) and p300, to activate gene expression. Many of the genes targeted by HIF-1 encode proangiogenic factors including VEGF-A (vascular endothelial growth factor A, abbreviated throughout this paper as VEGF) and EPO (erythropoietin) [1, 6, 7].

MicroRNAs (miRs) are endogenous, small, non-coding RNA molecules (~22 nt) that mediate gene expression at the post-transcriptional level. RNA polymerases II and III participate in the transcription of microRNA genes to produce miR primary transcripts (pri-miRs) that are usually several hundred nucleotides in length and contain conserved stem loops [8–10]. These pri-miRs are processed by the RNase III enzyme Drosha into stem-loop intermediates (~60–70 nt) which are termed precursor miRs (pre-miRs), and pre-miRs are actively transported out of the nucleus via a nucleocytoplasmic shuttler Exportin-5 (XPO-5) assisted by the GTP binding nuclear protein Ran [11, 12]. The pre-miRs in the cytoplasm are cleaved by another RNase III enzyme Dicer to become miR duplexes which are then incorporated into the miR-induced silencing complex (miRISC) [11]. Within the miRISC, proteins of the argonaute family (AGO) are essential for miR function in human as they facilitate the activation of miRISC by catalyzing the dissociation of miR guide strand (mature miR) from the passenger strand (cleaved later); only AGO1 and AGO2, among the eight AGO proteins in human, can mediate such strand dissociation during miR maturation [13, 14]. AGO1 is identified to be associated with miR-mediated translational repression; however, only AGO2-containing RISC is capable of catalyzing the cleavage of target mRNAs [15, 16]. Although previous research has extensively investigated the role of AGO in coordinating miRISC activities, very limited knowledge exists about the expression of AGO in response to cellular stress and its physiological importance in the remodeling of vasculature.

**Fig 1:**
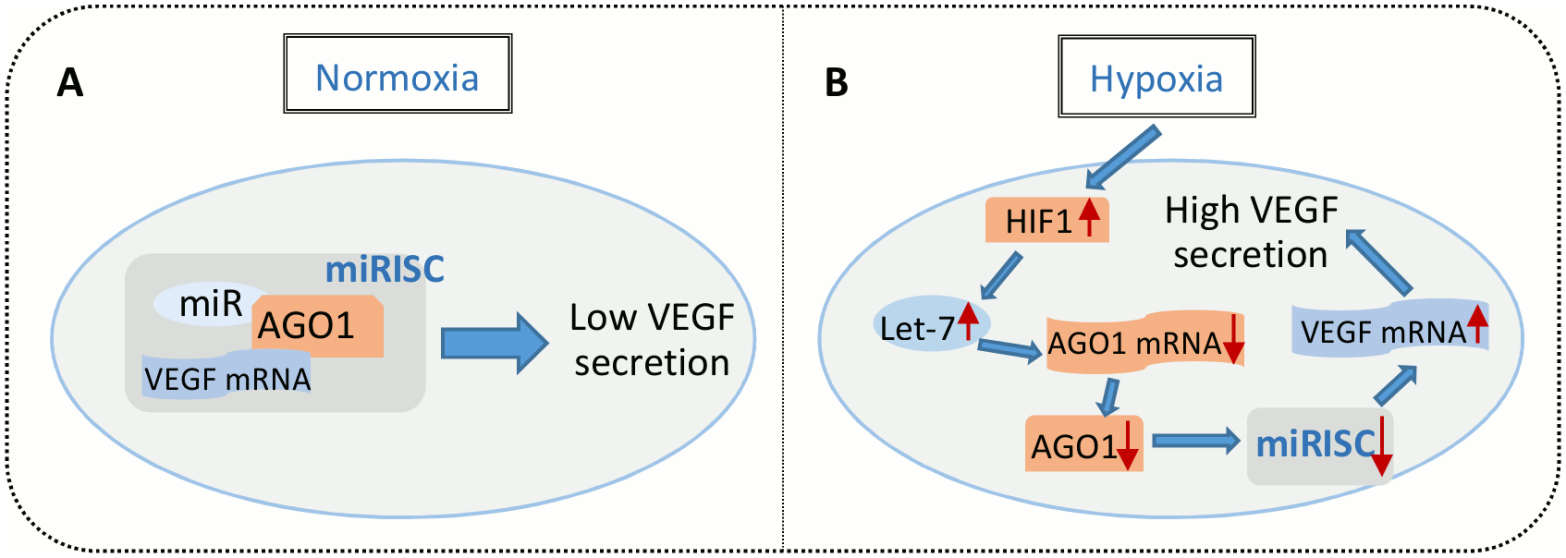
Translational repression of VEGF in normoxia and let-7 mediated VEGF desuppression in hypoxia in ECs. (A) VEGF mRNA were targeted by miRISC and inaccessible for translation in normoxia. (B) HIF-1 proteins that were stabilized in hypoxia induced let-7 biogenesis, which led to the downregulation of AGO1 mRNA, protein and miRISC formation. VEGF mRNA were desuppressed and ready for translation because of reduced miRISC activities.

Many miRs have been confirmed to have links with the pathophysiology of various cardiovascular diseases. Since endothelial cells (ECs) control the formation of new blood vessels (angiogenesis) which is critical for vascular homeostasis, dysfunction of ECs in response to adverse hemodynamic alterations and pathological stimuli, such as inflammation or chronic hypoxia, would lead to inadequate or anomalous angiogenesis that predisposes to the development of many vascular diseases including PAD (peripheral arterial disease) and CAD (coronary artery disease) [17]. MicroRNA let-7 (lethal-7) family is among the most promising miR candidates as novel regulators of angiogenesis considering its high expression in ECs and it directly targets several angiogenesis-related factors such as TSP-1 (thrombospondin 1), TIMP-1 (tissue inhibitor of metalloproteinases 1) and TGFBR1 (transforming growth factor beta receptor 1) [18–20]. A recent work by Chen et al. revealed that the HIF-1-let-7-AGO1-VEGF signaling pathway is essential in the control of EC angiogenesis in hypoxia [21]. Members of the let-7 miR family are identified as HRMs (hypoxia-responsive microRNAs) whose levels are robustly upregulated by HIF-1 transcription factor in hypoxia. Mature let-7 targets the mRNA of AGO1 and reduces the level of miRISC formed by AGO1 and other miRs that target VEGF, therefore freeing VEGF from translational repression to promote angiogenesis (Fig 1). Validated by *in vitro* and *in vivo* experiments, these findings supported the argument of an important angiogenic axis connecting HIF, miRs and AGO1 in ECs that may potentially serve as a valuable target for pro- and anti-angiogenic therapies [21].

Though many of the molecular components that are involved in the miR control of the HIF-VEGF pathway in ECs have been characterized, the detailed dynamics of how they mechanistically interact with each other within the signaling network are barely understood. In this sense, a computational model constructed from the perspective of systems biology would provide dynamic understanding and mechanistic insights of the complex cellular response to hypoxia, as the model relies on basic biophysical principles and biochemical reactions to describe relevant molecular interactions within a cell [22]. However, mathematical models of miRs are very limited in literature; the available models, such as the model of miR-193a in ovarian cancer and the model of miR control circuits in epithelial-mesenchymal transition, focused on predicting connections between certain expression patterns of miR-related molecules and disease-related physiological phenotypes [23, 24]. On the other hand, Kim et al. integrated the miR-451-mTOR signaling pathway into a multiscale hybrid model that described the complex processes of glioma cell proliferation and migration in great detail [25]. Most of these recent models have not considered time-course experimental data available from related studies in their validations and predictions, which may undermine the predictive power of computational models since any important details hidden in the dynamical responses would be easily overlooked. In this study, we have developed a mechanistic model describing the miR regulation of the HIF-VEGF signaling pathway that, for the first time at the molecular level, unveils the critical role of miR in the complex process of hypoxia-driven angiogenesis. The model incorporates biophysical details of miR biogenesis and considers cellular compartmentalization that were absent in previous miR pathway models. We have employed the model to study how different gene overexpression/silencing strategies in ECs would affect the overall cellular adaptation to hypoxia, quantitatively and qualitatively, by analyzing the dynamics of several signature proteins.

Assisted by the model, we have identified various characteristics in cellular oxygen sensing mechanisms and in miR regulatory network that controls the canonical HIF-VEGF pathway. The model predicts that HIF-1α stabilization obeys a hypothetical switch-like mode and it is negatively regulated by an mRNA destabilizer in hypoxia [26]. To address a major focus of the study, we show that let-7 and AGO1 are the initiators and coordinators of VEGF release, whereas they negatively exert feedback control on each other and are capable of minimizing the impact of possible outside perturbations; we also illustrate the role of miR-15a as a final effector molecule which is under control of AGO1, since abundance of miR-15a directly determines how much VEGF mRNA is available for translation. From these observations, we propose a potential mechanism that may contribute to impaired angiogenesis during recovery in patients with peripheral arterial disease. Another key focus of the study is the extension of our model analysis into potential clinical settings, where we evaluate different pathway-based therapeutic strategies designed to differentially regulate angiogenesis in highly hypoxic conditions, which are commonly observed in tumor and ischemic tissues. Together, these findings reveal an integrated image of multiple miRs, each with different targets, that work cohesively with miR-processing proteins (e.g. Dicer, AGO1) to counteract adverse physiological stresses by promoting VEGF synthesis and angiogenesis. This study should stimulate future research to investigate, both experimentally and computationally, the mechanistic signaling networks that contribute to the dysregulated miR expressions in cancer and in ischemic vascular disease.

## Results

### Formulation and assumptions of the model

**Fig 2:**
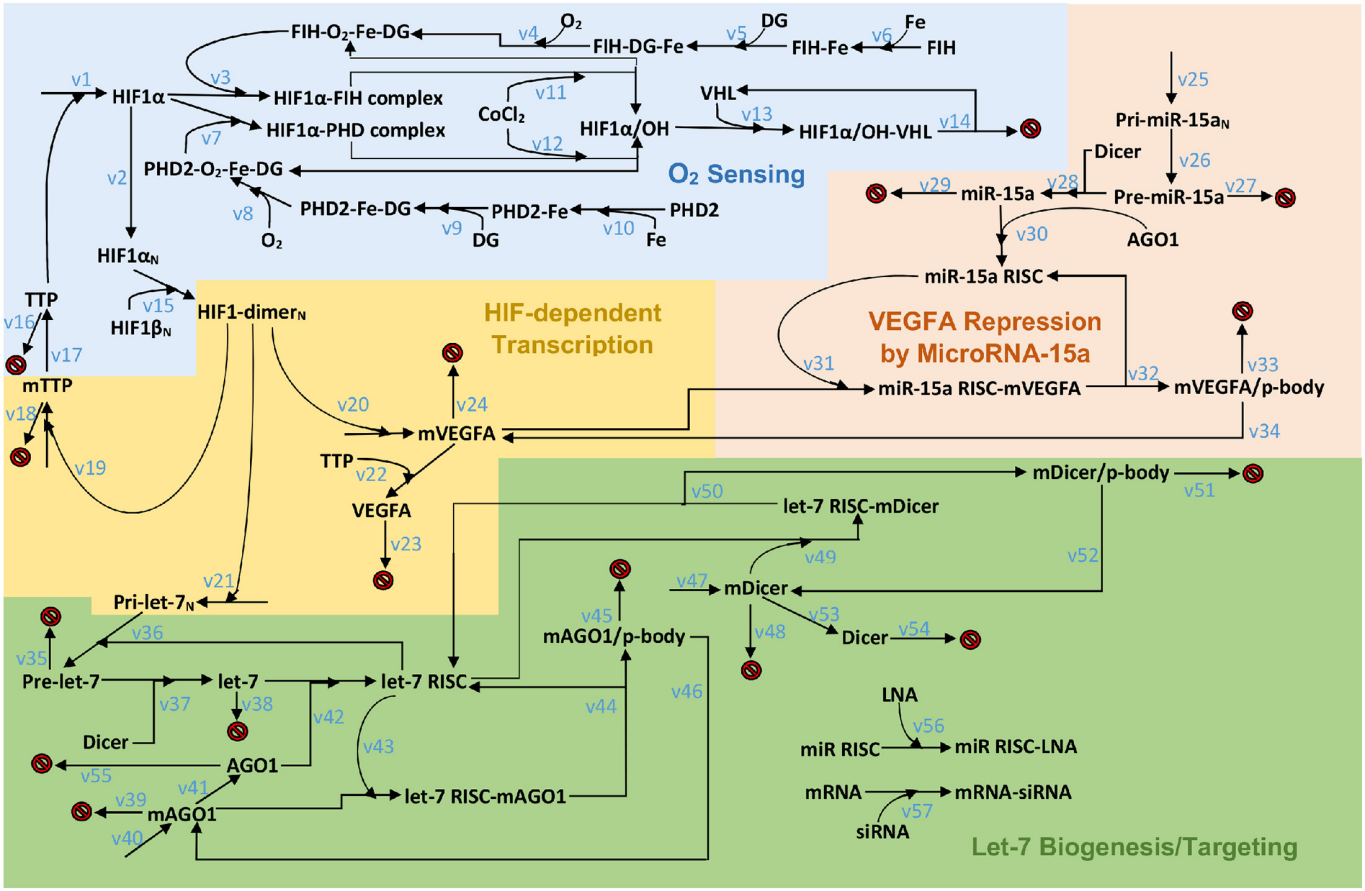
Proposed model scheme of the miR control of HIF-VEGF pathway. HIF-1α is stabilized in hypoxia and the HIF-1 dimer complex transcriptionally induces let-7 production. Mature let-7 represses AGO1 and leads to a global desuppression of VEGF. Model components in the colored backgrounds correspond to the four modules: blue/O_2_ sensing, pink/VEGF repression by miR-15a, orange/HIF-dependent transcription, green/let-7 biogenesis and targeting. Species whose names end with an N subscript are located inside the nucleus; reactions that point to red signs indicate degradation. The symbols v# refer to the 57 chemical reactions listed in S1 Table.

The model we constructed, as shown in Fig 2, describes the regulation and coordination by miRs in *in vitro* hypoxia-induced HIF stabilization and VEGF synthesis in ECs. The model consists of a cytoplasmic and a nuclear compartment, and it is functionally divided into four modules: oxygen sensing/HIF stabilization, HIF dependent gene transcription, miR-15a targeting of VEGF, and let-7 biogenesis/targeting. The oxygen sensing module is a selected integration of two established HIF models: Qutub and Popel’s work which considers the participation of iron and 2-oxoglutarate (2-OG) during HIF stabilization, and the model by Nguyen et al. that includes FIH-mediated events in HIF hydroxylation [27, 28]. Although the ascorbate binding suggested by Qutub and Popel as well as the process of nuclear HIF stabilization are not included in order to maintain a moderate complexity, our carefully integrated model is able to capture the essential oxygen-sensing behaviors of ECs that are needed to address our research focus. For the same reason we do not include the effects of reactive oxygen species and succinate on HIF-1α considered in subsequent papers of Qutub and Popel [29, 30]. The influence of HIF/PHD feedback is not considered since the model assumes that PHD2 concentration is in excess [31]. Although this feedback mechanism is absent, the model with the current parameter set and reactions is able to capture the core dynamics of the distinct HIF-1α behaviors in normoxia and in hypoxia; according to model simulations presented in this work, the high initial concentrations of PHD2 contributes to the early suppression of HIF-1α after its rapid induction in hypoxia, which agrees with an assumption made by Bruning et al. about the temporal role of HIF/PHD feedback loop [32]. To describe the negative feedback control of HIF in hypoxia, however, we included the mechanism of HIF-1α mRNA destabilization by TTP (tristetraprolin) identified by Chamboredon et al. and assumed a HIF-dependent TTP production, since hypoxic exposure is experimentally shown to induce TTP [33, 34]. Similarly, VEGF protein synthesis is also downregulated by TTP accumulated in hypoxia [35]. The module describing HIF activation of its targets, including the genes of VEGF and let-7, details the process of stabilized HIF-1α being transported into the nucleus, dimerizing with HIF-1β subunit and promoting the transcription of these genes containing HREs [21, 36]. We assumed that the step of HIF-1 complex binding with the coactivators CBP/p300 was included in the process of HIF-1α/HIF-1β dimerization. Interestingly, HIF-2α (hypoxia-inducible factor 2 alpha) is also shown by Chen et al. to transcriptionally induce the same group of HRMs upon its induction in hypoxia, and it is known that HIF-1α and HIF-2α share not only highly similar protein structures but also various common target genes including VEGF [21]. In skeletal muscle and especially ECs, HIF-2α signaling seems to be rather ancillary to the predominant regulatory potential of HIF-1α that primarily modulates the cellular angiogenic and migratory activities [37, 38]. Therefore, for the scope of this study we decided not to distinguish between these two molecules in the model but to represent them both in terms of HIF-1α, which is more prevalently expressed across different cell types than HIF-2α [39].

The model currently considers let-7 and miR-15a as key miR regulators of the hypoxia-driven VEGF desuppression process. The production of miRs in the model followed a well-established miR biogenesis pathway that undergoes transcription, nuclear-cytoplasmic transport, endonucleolytic processing and miRISC loading [11]. The model combines Drosha processing and XPO-5 transport into a one-step reaction, and miRISC formation along with miR duplex dissociation is simplified as one reversible association process between AGO1 protein and miR. The complex formed by AGO1 protein and let-7 can travel back into the nucleus and promote the processing of pri-let-7 which constitutes a positive auto-regulatory loop [40]. Let-7 represses the translation of two confirmed targets, AGO1 and Dicer, and this silencing negatively feeds back to the maturation and stabilization of let-7 [21, 41]. The mRNAs of AGO1 and Dicer are processed by the let-7 miRISC and directed to cytoplasmic domains called p-bodies [42, 43]. Since p-bodies are found to be involved in general mRNA turnover, we assumed that once mRNAs entered the p-bodies, they would be stored, inaccessible to translation with a significantly slower degradation rate compared to that of cytoplasmic mRNAs, while a very small fraction of them could still exit p-bodies and re-enter the translational machinery [44]. Since the let-7/AGO1 axis would influence the expression of a group of miRs leading to altered dynamics of many target genes, we selected VEGF, due to its crucial importance in angiogenesis and extensive literature data support, as an epitome gene to demonstrate mechanistically the details of how this cascade controls specific gene expression during a pro-angiogenic response. For the current purposes of the model, miR-15a is selected to represent a group of VEGF-targeting-miRs as miR-15a has been experimentally validated to directly repress VEGF synthesis and markedly affect angiogenesis in ECs [45]. In addition, hypoxia is shown to weaken the association of AGO1 with many VEGF-targeting miRs including miR-15a and cause significant downregulation of these miRs; these evidence further links the dynamics of miR-15a to the coordination by the let-7/AGO1 axis [21, 46]. VEGF mRNAs targeted by miR-15a also undergo a series of steps including p-body storage similar to the mechanism of let-7-mediated mRNA silencing. Details including the mathematical formulation of the biochemical reactions in the model and parameter optimization are discussed in the Methods section.

### Model validation

**Fig 3:**
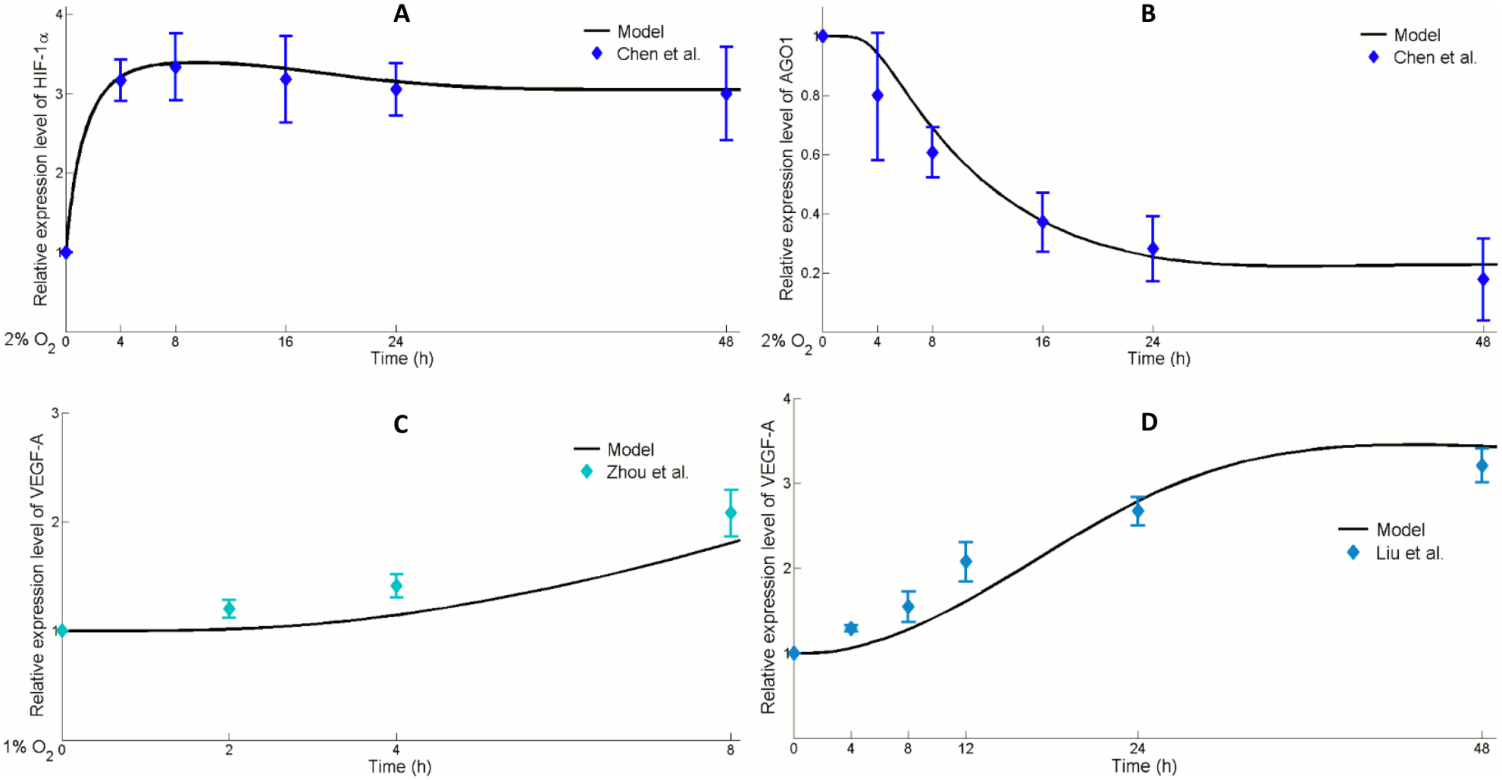
Comparisons between model simulations and experimental results. (A) HIF-1α protein is quickly induced and stabilized in 2% cellular O_2_ during a 48-hour simulation (line). (B) Hypoxia induces miR-mediated translational repression of AGO1 over time and reduces intracellular AGO1 protein abundancy (line). (A-B) Predicted time course expressions of HIF-1α and AGO1 are normalized and compared with the quantified Western blot data (symbol) in ECs with standard deviation values provided by Chen et al [21]. (C) 1% O_2_ induces VEGF protein production over 8 hours (line) in SHEP cells and (D) 200 μM of CoCl_2_ treatment in normoxia can mimic hypoxia in cells and induce VEGF protein synthesis in HepG2 cells during a 48-hour simulation (line). (C-D) We numerically quantified the two sets of raw VEGF Western blot data from literature in SHEP and HepG2 cells by densitometry (symbol), and the results with calculated standard deviation are compared with model simulations [50, 51].

The simulations of the model were compared with the data from independent experiments performed by different research groups. The first form of validation focused on the oxygen sensing module and compared the model’s prediction of HIF-1α accumulation in hypoxia with the quantified Western blot data in ECs (Fig 3A) [21]. Experimental data suggest that HIF-1α accumulation *in vitro* is most significant at O_2_ levels between 0.5–6%, and this is reflected in the model by setting the initial O_2_ concentration to 19.9 μM which corresponds to 2% ambient oxygen [27, 47]. The level of HIF-1α predicted by the model was the sum of both the free form and bounded proteins. HIF-1α concentration at time zero was taken as a reference measure which represents the normoxic (21% O_2_) steady state level. In agreement with the experimental result, the simulation showed a quick induction of HIF-1α during the first few hours followed by a gradual decrease to the steady state level. Similarly, Western blot data of HIF-1α in different cell types from other research groups also indicate that, in hypoxia, HIF-1α protein is induced rapidly while its expression peaks and gradually decreases after a few hours, suggesting that the cascade of TTP-mediated HIF-1α mRNA destabilization, as one of the major mechanisms which downregulate HIF-1α via negative feedback, should be incorporated as a fundamental part into the model [33, 48, 49].

In addition, the predicted time course abundance of AGO1 protein was compared with the experimental quantification using Western blot in ECs in hypoxia (Fig 3B) [21]. The predicted AGO1 level was also a summation computed in a similar way of how HIF-1α was defined above. To obtain the relative expression over time, the absolute level of AGO1 was normalized with respect to its initial concentration (steady state level in normoxia), which was obtained by simulating the model at an O_2_ concentration 209 μM (21% O_2_) for a long enough time span [27]. After an initial delay during which let-7 was accumulated in hypoxia and let-7 miRISC were formed, AGO1 level started to decrease because of a rapid decline in the amount of AGO1 mRNAs that were available for translation (S1 Fig). Chen et al. also demonstrated that this response was not specific to ECs: cells from different organs/tissues including liver, kidney and muscle all displayed significant AGO1 downregulation in response to hypoxia [21].

The VEGF protein production curve predicted by the model was compared with experimental data from two different groups. Although endothelial cells, compared to other cell types, may not be the biggest contributor of VEGF secretion in response to low oxygen tension, adequate autocrine VEGF signaling was proven to be critical in the maintenance of vascular homeostasis [52]. Zhou et al. measured the VEGF protein expression at different time points in whole-cell extract of SHEP cells (a human neuroblastoma cell line) that were cultured in hypoxic conditions (1% O_2_) [50]. A two-fold increase in VEGF level was predicted after a simulated 8-hour exposure to 1% O_2_ tension (Fig 3C). Liu et al. analyzed the impact of CoCl_2_-induced hypoxia on the expression of VEGF proteins using Western blot in HepG2 cells (a human hepatocellular carcinoma cell line) [51]. CoCl_2_ (cobalt chloride) is one of the hypoxia-mimetic agents; it stabilizes HIF-1α in normoxia by directly inhibiting the process of PHD/FIH-mediated HIF-1α hydroxylation (see reactions in Fig 2) [53, 54]. The model assumes an initial CoCl_2_ concentration of 200 μM in the simulation and the relative expression was normalized to VEGF level at time zero (Fig 3D). Since the current model reactions and parameters are established to describe signaling events specifically in ECs, the fit between model predictions and experimental results in Fig 3C and 3D does not imply that ECs, HepG2 and SHEP cells have the same dynamics of intracellular signaling and VEGF production. Likely, different parameter sets would be needed in order for the model to make more accurate predictions of VEGF synthesis in other cell types (e.g. tumor, muscle, stromal cells) as they are the more significant sources of VEGF secretion compared to ECs [55]. Previous studies have quantified the induction of VEGF in stromal and tumor cells and found a 2 to 6 fold increase of VEGF in stromal cells and a 3 fold increase in a breast cancer cell line after 24 hours of hypoxia treatment; our EC-based model predicts a 3.5 fold increase in the intracellular VEGF level after 24 hours of simulation at 2% O_2_ (S2C Fig) [56, 57]. In this sense, the model described in this work is able to predict VEGF dynamics that are comparable, both quantitatively and qualitatively, to biological VEGF data in response to hypoxia in different types of cells, which allows for further extension of the HIF-let-7-AGO1-VEGF framework in other cell models.

### Oxygen sensing and graded profiles of HIF-1α

**Fig 4:**
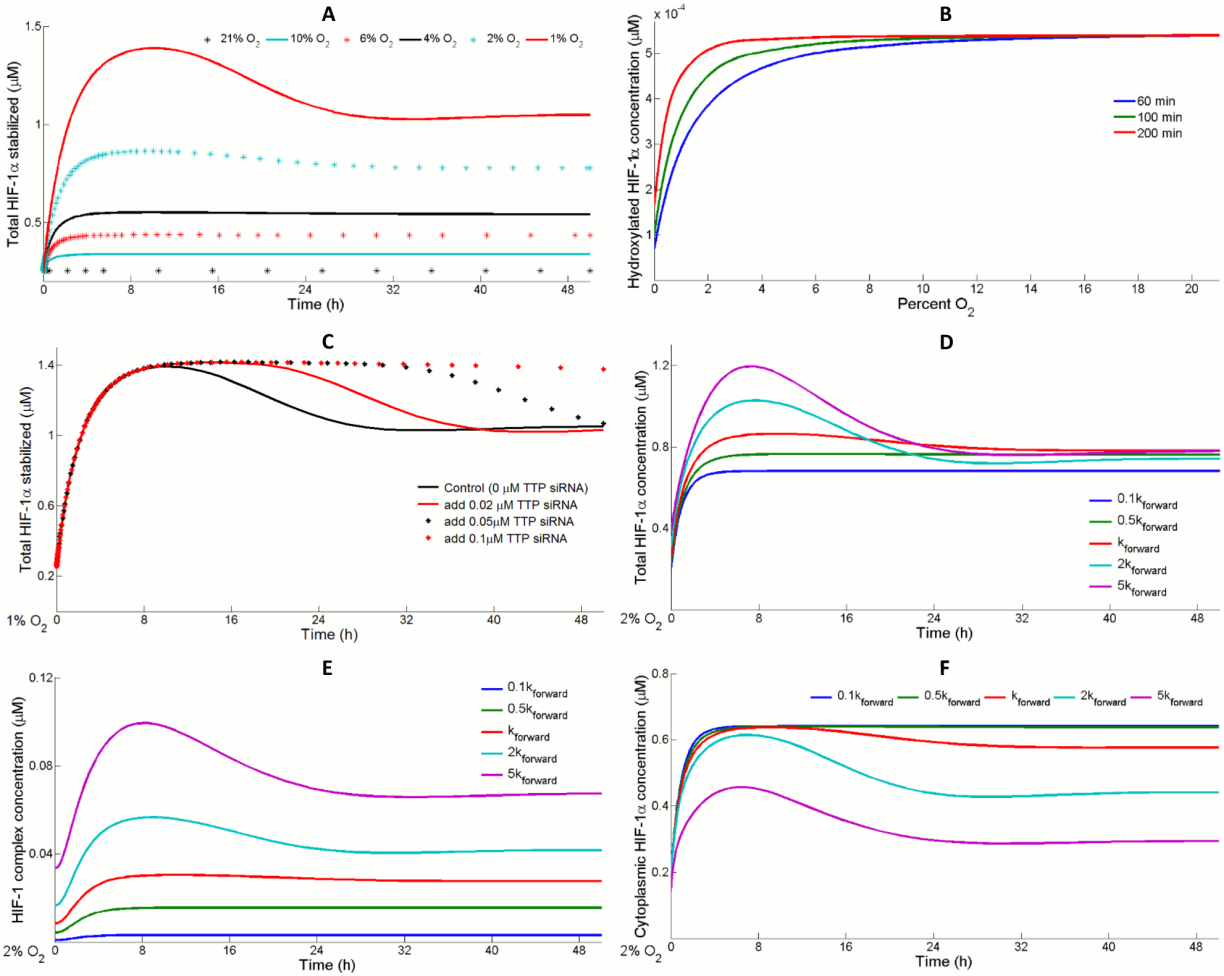
HIF-1α synthesis in hypoxia. HIF-1α level at time zero is the steady state normoxic (21% O_2_) level. (A) Total HIF-1α expression profiles are highly sensitive to oxygen availability. (B) When PHD2 initial concentration is in excess, an oxygen-dependent, switch-like behavior in the amount of hydroxylated HIF-1α is observed. As simulation span increases, a steep reduction in HIF-1α hydroxylation occurs between 2% to 4% O_2_. (C) TTP is responsible for the delayed drop (initial overshoot) in the induction of HIF-1α in hypoxia. By increasing the dose of a simulated siRNA that silences TTP expression (assuming siRNA binds TTP mRNA potently with a K_d_ of 1 nM), the duration of the initial overshoot is lengthened. (D) Varying the rate of HIF-1α import from cytoplasm into nucleus (k_forward_) affects the overall HIF-1α profile in hypoxia. (E) Larger k_forward_ values of HIF-1α nuclear import result in higher levels of HIF-1 transcription factor complex formed. (F) Smaller k_forward_ values lead to lower total HIF-1α levels in normoxia and in hypoxia, while the majority of induced HIF-1α is located only in the cytoplasm and unable to form transcription complex with HIF-1β. (D-F) Magnitude of k_forward_ is set to 10%, 50%, 200% and 500% of its original value respectively in the comparisons. For each k_forward_ value, steady state levels of all species, after the model is simulated in normoxia for a long time span, are collected and considered a new set of initial conditions.

The step of oxygen sensing determines how much HIF-1α will be stabilized and then dimerize with HIF-1β to form active transcription factors at different O_2_ levels. Initially, HIF-1α expression is low in normoxia and transcriptional activities of let-7, VEGF and TTP are insignificant. As oxygen availability decreases, hydroxylation of HIF-1α by PHDs and FIH is also reduced, allowing more HIF-1α to escape from VHL-mediated degradation and enter the nucleus [1]. Fig 4A shows the overall oxygen dependent response of HIF-1α. For small enough oxygen levels 2% and 1% in Fig 4A, accumulated HIF-1 reaches a maximum (overshoot) at around 10 hours and then slowly declines to a steady level. Since the model assumes that initial PHD2 concentration is in excess in order to capture switch-like responses in HIF-1α hydroxylation (Fig 4B), changes in PHD2 dynamics when hypoxia is sensed should take place very quickly at a time point much earlier than the overshoot [26, 27]. The same reason justifies that FIH does not cause the overshoot, so we hypothesize that it is TTP which creates the initial overshoot, since hypoxia promotes TTP synthesis which destabilizes the mRNAs of HIF-1α and downregulates its translation [34]. Results in Fig 4C show that *in silico* knockdown of TTP mRNA effectively prolongs the initial overshoot in HIF-1α stabilization curves. The time course profiles of HIF-1α also strongly depend on the rate of cytoplasmic-nuclear trafficking (Fig 4D). As the forward rate of HIF-1α shuttling from cytoplasm into nucleus increases, more HIF-1 transcription factor complex is formed, which promotes the synthesis of various molecules including VEGF that facilitates cellular adaptation to hypoxia by improving angiogenesis and TTP that feeds back to inhibit HIF-1α production (Fig 4E). Consistent with our prediction, Ahluwalia et al. overexpressed importin-α, a nuclear importer of HIF-1α, in GMECs (gastric mucosal endothelial cells) of aging rats and observed a significant increase in the binding of HIF to the VEGF gene promoter region [58]. Smaller forward rate of HIF-1α nuclear import leads to a lower HIF-1α baseline in normoxia and reduces the overall HIF-1α level in hypoxia, since the majority of the stabilized HIF-1α accumulates within the cytoplasm (Fig 4F), where HIF-1α is unable to dimerize with HIF-1β and is more susceptible to degradation. This suggests that cells with impaired HIF-1α nuclear import are correlated with reduced HIF-1 transcription activity and poorer pro-angiogenic adaptation in hypoxia, which is consistent with the finding that reduced importin-α level in senescent GMECs hindered the induction of VEGF expression and angiogenesis in response to hypoxia [58].

### Let-7 and AGO1 coordinate downstream VEGF desuppression by modulating the silencing activity and availability of miR-15a

We are interested in the mechanistic interactions between let-7, AGO1 and miR-15a and their roles in the control of subsequent VEGF mRNA release, since AGO1-associated miRs (e.g. miR-15a) that are capable of silencing VEGF were shown to be less abundant following hypoxia treatment [21]. To better understand this key feature in our model, we approach the analysis in two steps by looking at the direct interactions between let-7 and AGO1 that influence free form VEGF mRNA level at different O_2_ tensions, as well as the downregulation of miR-15a availability as a result of upstream let-7/AGO1 control.

#### Let-7/AGO1 feedback loop and upregulation of VEGF translation

**Fig 5:**
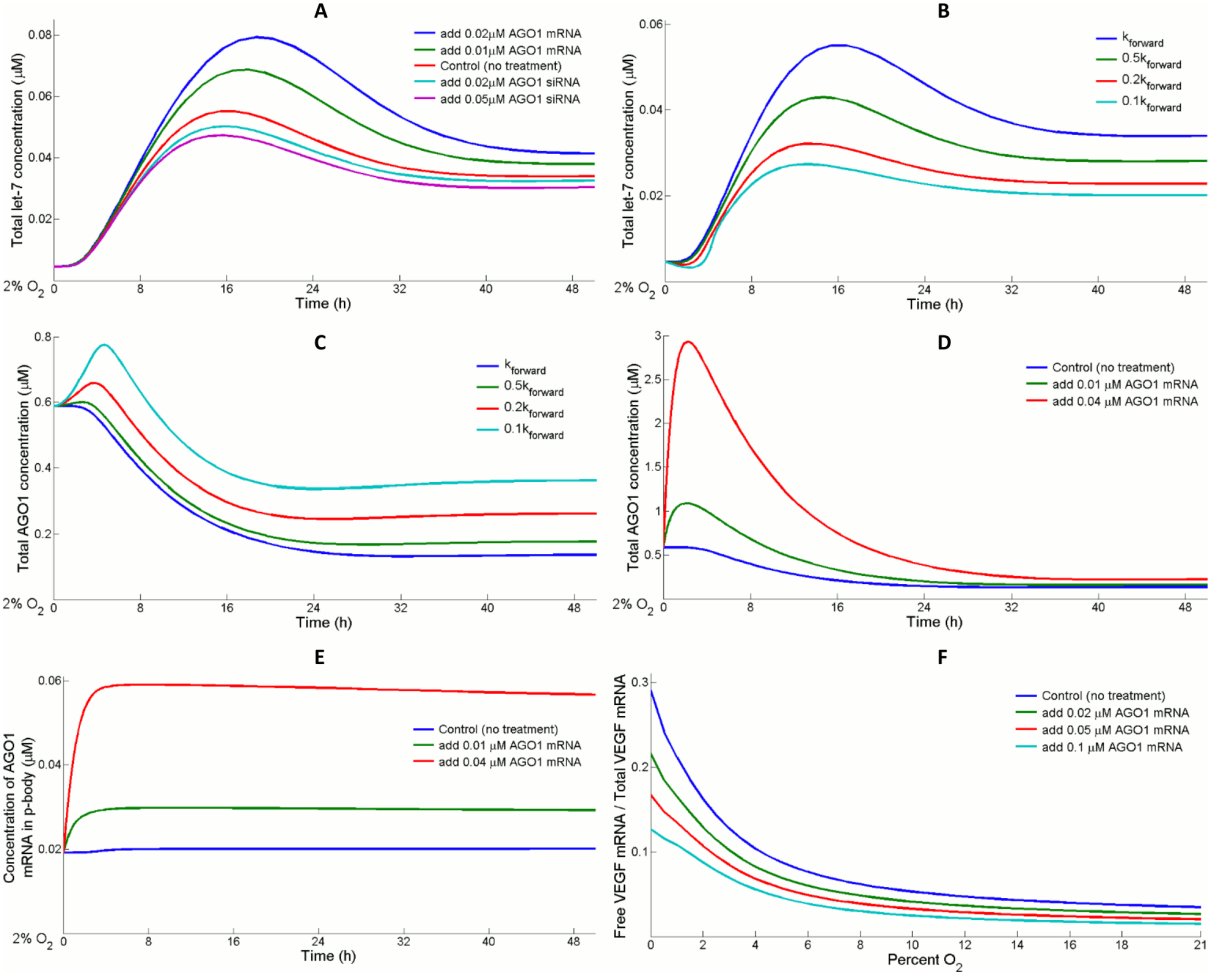
Let-7 and AGO1 mutually control each other in hypoxia. (A) Varying the cellular AGO1 abundance by antagonizing or overexpressing its mRNA changes the let-7 availability. (B) Let-7s that are in association with AGO1 are less prone to degradation, so a decrease in the binding strength of let-7 toward AGO1 causes more let-7 to be degraded. Consequently, (C) let-7-mediated activity including AGO1 repression is downregulated, allowing additional AGO1 protein synthesis. (B-C) Association rate of AGO1 and let-7 (k_forward_) is adjusted to 10%, 20%, and 50% of its original value respectively in the comparisons. (D) AGO1 overexpression leads to an early upstroke in its time course profile but its steady state level changes insignificantly. (E) After 4 hours, almost all the additional AGO1 mRNAs (e.g. 0.01 μM and 0.04 μM) being introduced in the beginning are fully shuttled into the p-body to become inaccessible for translation. (F) In hypoxia, VEGF mRNA released from miRISC, in combination with HIF induction, boosts the pool of free form VEGF mRNA. A simulated AGO1 overexpression rescues the drop in miRISC level and drives free form VEGF mRNA back into miR-mediated repression. (A, D-F) The model assumes that in AGO1 silencing, siRNA binds AGO1 mRNA potently with a K_d_ of 1 nM; AGO1 overexpression is simplified as an increase of certain amount in the initial concentration of AGO1 mRNA.

During hypoxia, accumulated HIF-1 complex transcriptionally activates the production of let-7 [21]. Mature let-7s are then loaded into the RISC which is simplified as a one-step association with AGO1 in the model. The steady-state levels of let-7 and AGO1 in normoxia are in a dynamic equilibrium: AGO1 is in large excess over the demand for let-7 RISC formation. Since AGO1 mRNA is validated as a target of let-7, when cellular condition changes from normoxia to hypoxia, let-7 expression is robustly induced which breaks the previous balance and leads to a downregulation of AGO1 [21]. AGO1 would only drop to a certain level before it starts to manifest its negative feedback control on let-7, and the system shifts to a new steady state (hypoxia) for both species. The predicted trend of let-7 expression (Fig 5A, red curve) matches the experimental result, showing that mature let-7 is induced in the beginning but then drops gradually during a 48-hour hypoxic treatment (S2A and S2B Fig) [21]. From a biological perspective, it is believed that free form miRs that are unassociated with AGOs are less resistant to degradation, which in this scenario suggests that AGO1 expression is essential in the stabilization of let-7 [59]. Therefore in hypoxia, enforced silencing/overexpression of AGO1 is predicted to reduce/enhance let-7 expression (Fig 5A). By the same reasoning, a decrease in the binding affinity of let-7 toward AGO1 would lead to fewer total let-7 because of increased miR degradation (S3 Fig), which would then give rise to a higher AGO1 level at steady state since let-7-mediated translational repression is reduced (Fig 5B and 5C). On the other hand, increasing the initial concentration of AGO1 mRNA causes a significant short-term rise in its expression; however, it only induces a negligible change in its steady state, since overexpression of AGO1 inhibits its own translation as let-7 mediated mRNA repression becomes stronger (Fig 5D). Fig 5E reveals that after 4 hours, by measuring the differences between the red/green and blue curves, nearly all of the additional AGO1 mRNAs being introduced initially are transferred to the p-body where they are translationally repressed; at the same time, AGO1 protein expression starts to decline.

In hypoxia, a large percentage of VEGF mRNAs that were suppressed by miRISCs in normoxia are released because of the downregulation in AGO1, plus the HIF-1-induced portion, constitute the total VEGF mRNA pool available for translation. Consistent with the hypothesis of Chen et al. that miR-mediated VEGF translational desuppresion is crucial to the elevated VEGF synthesis in hypoxia, the model predicts that the ratio of free form VEGF mRNA to total VEGF pool decreases as O_2_ availability increases, and AGO1 overexpression depresses the percentage of free form VEGF mRNA in hypoxia (Fig 5F), resulting in decreased VEGF production which agrees with published experimental results (S4 Fig) [21].

#### MiR-15a expression is controlled by AGO1 and Dicer availability during hypoxia

**Fig 6:**
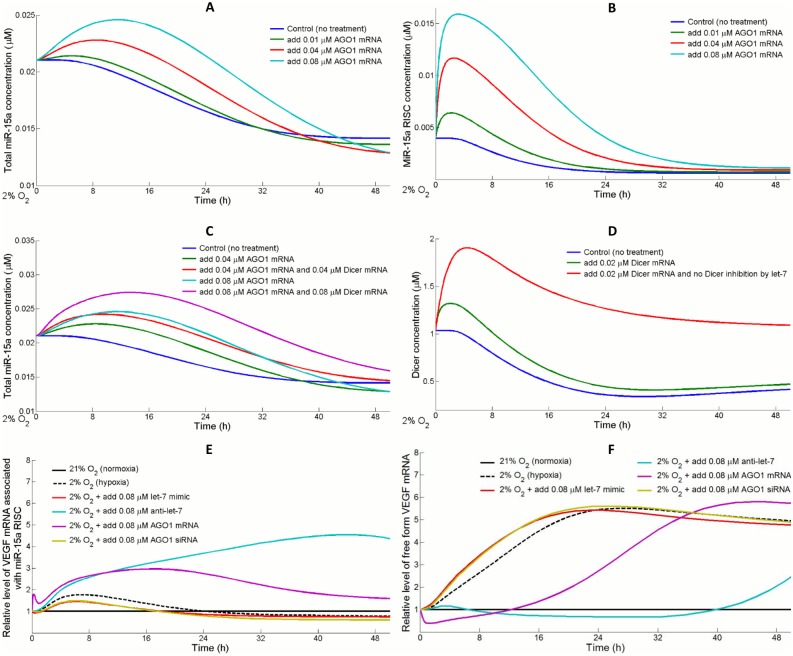
Let-7 represses Dicer and AGO1 that both limit miR-15a expression in hypoxia. (A) Overexpression of AGO1 mRNA by 0.01, 0.04 and 0.08 μM in hypoxia leads to short-term rises in the expression levels of total miR-15a (B) by promoting the association of free form miR-15a with AGO1 to make more miR-15a RISCs. (C) Dicer processing is a limiting step in the production of miR-15a in hypoxia. Introducing both Dicer and AGO1 mRNAs at the beginning of simulation results in elevated miR-15a abundance compared to adding AGO1 mRNA alone. (D) When let-7 no longer inhibits Dicer translation, an overexpression in Dicer mRNA generates a remarkable change in the expression profiles of Dicer with respect to the control situation. With let-7 mediated Dicer silencing, the response of Dicer mRNA overexpression is significantly attenuated. (E) Relative expression of non-translatable VEGF mRNA associated with miR-15a RISC and (F) translatable VEGF mRNA in response to different treatment strategies. Hypoxia causes an initial increase in the binding between VEGF mRNA and miR-15a RISC because of the rapid HIF-1-activated VEGF transcription, but the impact of AGO1 silencing becomes dominated later that, in the long run, miR-15a-bound VEGF is reduced compared to the normoxic level. In hypoxia, enforced let-7 overexpression or AGO1 silencing modestly increases the amount of translatable VEGF, while let-7 antagonists or AGO1 overexpression can remarkably blunt VEGF production.

MicroRNA-15a is one of the potent anti-angiogenic miRs that inhibit vascular growth, and it is found to directly repress VEGF in ECs and in cancer cells [45, 60]. For this reason, other miRs that were previously identified to target VEGF in ECs (e.g. miR-200b) are also referred as miR-15a in the model for simplicity [61]. Similar to the shielding effect that stabilizes let-7, overexpression of AGO1 temporarily elevated the formation of miR-15a RISC and total miR-15a levels in hypoxia (Fig 6A–6B). Unlike let-7, the model assumes that transcription of miR-15a is not affected by hypoxia, so it is reasonable to hypothesize that the downregulation of miR-15a is partially attributed to the availability of Dicer, which is predicted to be significantly downregulated by let-7 in hypoxia. Many groups have discovered different underlying mechanisms that contribute to the silencing of Dicer in hypoxia in various cell types [62–64]. Our model proposes another reasonable explanation to Dicer silencing: hypoxia strongly induces let-7 which directly targets Dicer for translational repression, and this mechanism works in accordance with let-7-AGO1 pathway to cut down miR biogenesis in hypoxia. A combination of Dicer and AGO1 overexpression moderately increases the level of mature miR-15a, compared to AGO1 overexpression alone (Fig 6C). Given that translation of Dicer is strongly inhibited by let-7, Dicer protein expression quickly drops to a low state after the initial burst after enforced overexpression, while in another situation Dicer is expressed more persistently upon the removal of the inhibitory effect (Fig 6D). As mentioned earlier, the desuppression process of VEGF from the regulation of miR-15a is mediated by the expressions of let-7 and AGO1 in hypoxia. Our analysis reveals that the amount of VEGF mRNAs that are incorporated into miR-15a RISC and thereby subjected to translational repression is highly influenced by the abundance of let-7 and AGO1 over time (Fig 6E); both strategies, antagonizing let-7 or overexpressing AGO1 in hypoxia, can drive a much greater volume of newly synthesized VEGF mRNA toward the state of miR-15a-mediated repression compared to hypoxic control conditions. This would result in a markedly delayed rise in the level of intracellular free form VEGF mRNAs that are ready to go through translation (Fig 6F), suggesting that the effect of hypoxia-driven VEGF synthesis would be much less notable.

### Therapeutic strategies target the HIF-miR-AGO-VEGF pathway to control angiogenesis

A number of *in vitro* and *in vivo* studies have been performed to characterize the therapeutic values of different miRs in treating cancer and cardiovascular disease; in some of these the goal was to uncover the entire regulatory events that give rise to the miR dysregulation [20, 65, 66]. Abnormal profiles of AGOs that disrupt the balance between pro- and anti-angiogenic miR expression are among the essential reasons behind the aberrant disease-related angiogenic activities of ECs [67, 68]. Deriving and testing potential miR-based therapeutics in different diseases *in silico*, given the substantial analysis performed to understand our proposed pathway, are of crucial significance to future research in the field.

#### Tumor

**Fig 7:**
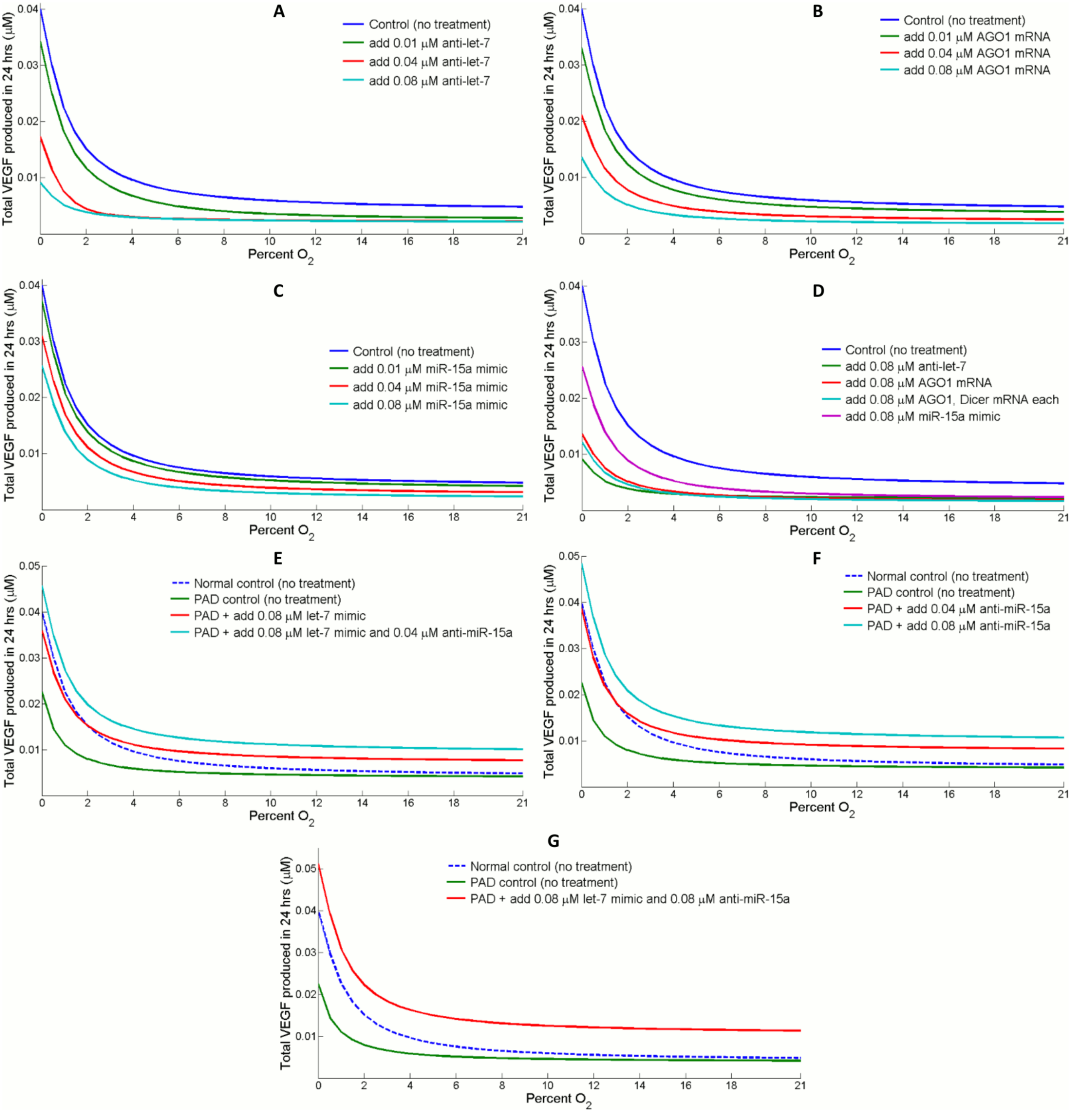
Simulations of potential pathway-related therapeutic strategies in tumor and PAD. Different doses of (A) let-7 antagonists, (B) AGO1 mRNA overexpression, and (C) miR-15a mimics are applied and total VEGF production curves at different O_2_ tensions are obtained. (D) Either antagonizing let-7 or overexpressing AGO1 strongly suppresses VEGF synthesis compared to the control curve, particularly in severely hypoxic conditions (0–2% O_2_) that simulate *in vivo* tumor oxygenation. More specifically, let-7 antagonist is more effective because it maintains a stable and higher intracellular AGO1 expression than direct AGO1 overexpression. Since both AGO1 and Dicer are significantly reduced in hypoxia and are limiting factors in miR maturation and stabilization, the response of miR-15a overexpression is relatively insignificant. The results should be evaluated qualitatively since many proteins could have much lower concentration *in vivo* compared to *in vitro* cultures which might invalidate some of the model assumptions. (E-F) Testing the impact of different doses of let-7 mimics and miR-15a antagonists on VEGF release in PAD conditions, which is modeled as a weak let-7 induction by HIF-1 in hypoxia (see Methods for details). Antagonizing miR-15a, compared to overexpressing let-7, results in stronger promotion on VEGF production. (G) The combination of miR-15a antagonist with let-7 mimic positively modulates angiogenesis; it elevates VEGF production by more than two fold with respect to PAD controls in hypoxia.

Two independent studies have linked high AGO2 expression with enhanced myeloma angiogenesis and high risk in multiple myeloma prognosis [69, 70]. On the contrary, low AGO1 expression in combination with high let-7 and high VEGF profile are a set of markers observed in the hypoxic core regions of mouse tumor xenografts; in addition, statistical analysis has revealed that HCC (human hepatocellular carcinoma) patients with low AGO1 and high VEGF levels are correlated with poor cancer survival [21]. Regarding this issue, we proposed and evaluated four different approaches, with the help of the model, to inhibit VEGF synthesis in tumor: (1) direct antagonizing of let-7, (2) overexpression of AGO1 mRNA, (3) overexpression of AGO1 and Dicer mRNA, (4) overexpression of miR-15a by miR mimics. Simulations in Fig 7A–7C compare the total amount of VEGF produced during a 24-hour span in the control condition and in the treated condition. Fig 7D summarizes the impact of all four approaches and suggests that both AGO1 overexpression and let-7 antagonizing effectively decrease the synthesis of VEGF, especially in hypoxia. Although tissues *in vivo* have a wide spread of oxygen levels, 21% O_2_ (*in vitro* normoxia) is still multiple fold higher than the *in vivo* oxygen tension in most arterial beds, and tumors grow in even more hypoxic conditions where O_2_ level around 1% sets the border between adequate and poor oxygenation [71]. Nevertheless, in 0–2% O_2_ conditions that mimic *in vivo* tumor oxygenation, antagonizing let-7 or overexpressing AGO1 forcefully inhibits VEGF synthesis by a consequent three to four-fold decrease in total VEGF produced (S5 Fig). While an extra amount of Dicer, as demonstrated earlier, does promote miR-15a stabilization on top of the influence of AGO1 overexpression, it fails to exert a notable impact on VEGF inhibition in this scenario. This could be due to the sequential attenuation of Dicer’s influence in the cascaded reactions that ultimately suppresses VEGF. Interestingly, the fourth approach which is introducing miR-15a mimics, has only an insubstantial effect on the inhibition of VEGF production despite the fact that miR-15a directly targets VEGF. The observation that AGO1 reduction in hypoxia quickly destabilizes miR-15a is likely a major reason. Besides, miR-15a mimics available are essentially equivalent to precursor miRs which are subjected to subsequent Dicer cleavage; since Dicer is also downregulated in hypoxia, only a fraction of miR-15a mimics can be processed into mature and functional miRs.

#### Peripheral arterial disease

Given the growing importance of miRs in the development and recovery of several cardiovascular diseases, we would like to explore the potential application of our computational model in ischemia-related diseases, particularly in peripheral arterial disease [65, 72, 73]. Stather et al. discovered that certain miRs in the circulating blood plasma express differentially in PAD patients compared with controls, and members of let-7 family were among the top candidate miRs that were downregulated in the PAD group [74]. Motivated by this finding, we propose that in ischemic tissues, the downregulation of let-7 leads to higher AGO1 level and weakens the pathway of AGO1-mediated VEGF release as described in our model, thereby resulting in impaired angiogenesis and hindered perfusion recovery in PAD patients. Since the reason behind let-7 downregulation in PAD is unclear, to account for this observation the model assumes that HIF-1 induction of let-7 transcription in ischemic condition is much weaker than in non-PAD condition. By quantifying the total amount of VEGF produced during a 24-hour span, the model simulates and compares dose-responses of two therapeutic approaches, overexpressing let-7 and antagonizing miR-15a, which we proposed to enhance VEGF synthesis in PAD (Fig 7E and 7F). In this case, miR-15a build-up and increased VEGF suppression are the final outcomes of let-7 downregulation in PAD, so immediate VEGF release because of miR-15a loss-of-function is more promising than indirect strategies such as overexpressing let-7 or silencing AGO1 (S6 Fig). Even in extremely hypoxic conditions, combined miR therapeutics are shown to effectively restore (and even promote) VEGF production in ischemic tissues compared to controls (Fig 7G).

### Sensitivity analysis of pathway signature molecules

**Fig 8:**
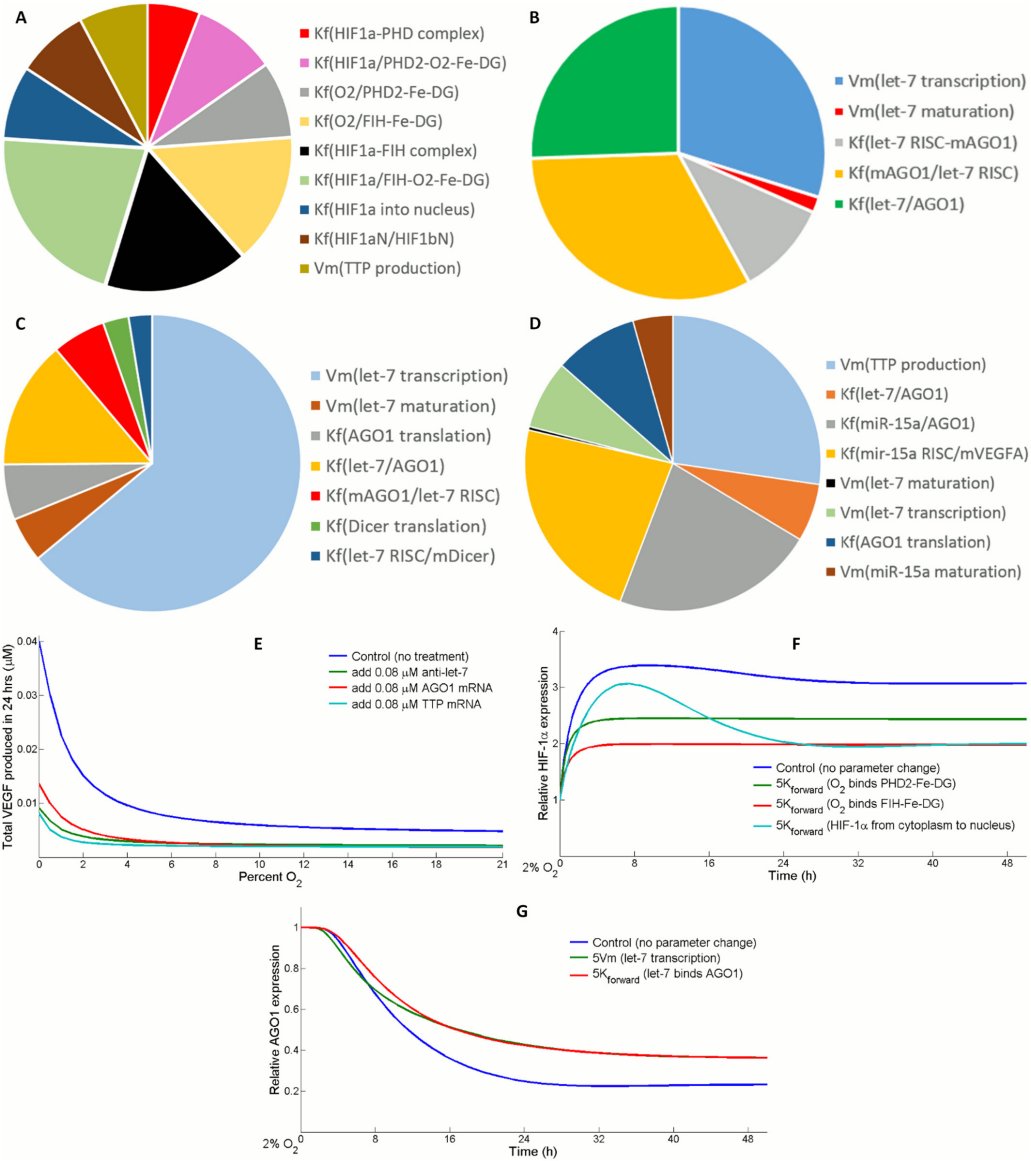
Sensitivity analysis of key species in the pathway. Sensitivity of (A) cytoplasmic HIF1-α, (B) free form AGO1, (C) free form let-7, and (D) VEGF to variations in different sets of kinetic parameters (direct production and degradation rates excluded). (A-D) Kf(X/Y) stands for the forward reaction constant of species X binding species Y; Vm(X) stands for the speed of reaction X; Kf(X) stands for the forward rate constant of species X dissociation. Detailed description of each parameter is available in the supplemental information. (E) At different O_2_ levels, TTP mRNA overexpression is tested as an anti-angiogenic therapy *in silico* compared to miR-based therapeutic strategies. (F) Affinity of O_2_ binding with PHD2 or FIH and HIF-1α translocation rate contribute to the trend of HIF-1α stabilization in hypoxia. (G) Relative downregulation of AGO1 in hypoxia is influenced by its binding with let-7. (F-G) Parameters are set to 500% of their original values in the comparisons. For each new value, steady state levels of all species, after the model is simulated in normoxia for a long time span, are collected and considered a new set of initial conditions. HIF-1α and AGO1 levels in hypoxia are normalized with respect to their concentrations at time zero (normoxic steady states).

We performed modular sensitivity analysis on four key species in the pathway: cytoplasmic HIF-1α, free form AGO1, free form let-7 and VEGF. The analysis was accomplished in MATLAB SimBiology toolbox (see Methods), assuming 2% hypoxia as O_2_ initial condition. Fig 8A–8D display the local sensitivity of the four species with respect to different set of selected kinetic parameters, in which direct production and degradation rates of each species were excluded since their contributions are too evident to produce valuable insights. Not surprisingly, HIF-1α is very sensitive to its affinity with PHD2-O_2_-Fe-DG or FIH-O_2_-Fe-DG complexes which will subsequently mark it for hydroxylation (Fig 8A). Also, the idea we proposed in previous results that the strength of association between let-7 and AGO1 controls the expression of both molecules, was again corroborated by sensitivity analysis (Fig 8B and 8C). It is noteworthy that in the sensitivity analysis for VEGF, varying TTP synthesis was as influential as varying the VEGF silencing efficiency of miR-15a RISC directly (Fig 8D). Since the major conclusions of this work relate closely to the qualitative dynamics of AGO1 and VEGF, the more influential parameters identified from modular sensitivity analysis were subjected to additional evaluations (S7 Fig). Motivated by the sensitivity analysis, we suggested that overexpressing TTP could be a potential anti-angiogenic therapy since it inhibits VEGF synthesis by decreasing HIF-1 signal and directly destabilizing VEGF mRNA. Since TTP suppression in cells has been associated with pro-tumorigenic phenotypes, stimulation of its expression might be an effective way to shrink the synthesis of pro-angiogenic (e.g VEGF) and pro-inflammatory cytokines in tumor (Fig 8E) [75, 76].

In addition to the sensitivity analysis, we tried to look for core reactions and parameters that are responsible for the radical differences between system behaviors in hypoxia and in normoxia. HIF-1α and AGO1 are both master coordinators for a series of signaling events in the model, and their time course total expressions have been experimentally measured [21]. Increasing the affinity between oxygen and PHD2-Fe-DG or FIH-Fe-DG complex significantly reduces the relative HIF induction in hypoxia (Fig 8F). A reason for that is the extra amount of PHD2/FIH-Fe-DG-O_2_ that has been stabilized in normoxia due to enhanced O_2_ binding, which speeds up HIF hydroxylation in hypoxia (S8 Fig). A higher rate of HIF-1α translocation also strongly influences HIF induction because it temporarily pushes more HIF-1α into the nucleus and protects it from degradation, but this would later lead to a big drop in total HIF-1α because of HIF-induced TTP production (Fig 8F). For AGO1, its downregulation is necessary to induce sufficient VEGF desuppression. Like let-7, in the model AGO1 is prevented from degradation when associated with miRs. Since more let-7-AGO1 is formed because of either increased let-7 or stronger binding, the extra amount of AGO1 that is stabilized elevates its baseline level in hypoxia; furthermore, higher level of let-7-AGO1 reduces AGO1 level in normoxia due to translational repression (Fig 8G).

## Discussion

In this study, we presented the first mass-action based computational model of miRs in a comprehensive, whole-cell signaling network that is closely related to angiogenesis. Our model is also the first one to have described in details the regulatory process that controls miR biogenesis, such as Dicer cleavage, AGO1 loading and p-body localization, and considered these reactions as an essential module of the model framework [11, 42]. The reason why these elements are important in the formulation of our model is more than the fact that they represent the real biological process; for example, AGO1 is commonly considered as part of miRISC for universal miR-mediated silencing activities, but in our pathway of interest it also happens to be a key factor. Challenges follow as always when researchers map signal transduction pathways, and we aim to make a model that contains enough biochemical and biophysical details to address different experimental findings while its complexity remains manageable. With a few Michaelis-Menten or Hill kinetics and more than 80% of the reactions based on simple first and second order kinetics, the model is equipped with both modular flexibility and solid biochemical background. Since most miR research only focuses on the influence of one specific miR, we decided to take a novel approach and investigate the miR-dependent regulation of miRs in hypoxia coordinated by miR-processing molecules AGO1 and Dicer, as the core theory of our model has been experimentally validated by the work by Chen et al [21]. However, current experimental data on miR signaling in the control of angiogenesis is very thin, which leaves a large space for our model to be further validated and refined. A fundamental goal of this work is to guide and stimulate more miR research that would investigate not only the function of an individual miR but also the interconnections between abnormal profiles associated with a group of miRs in vascular diseases. By the same reasoning, topics such as the time course dynamics of molecules participating in p-body configuration during miR-induced mRNA silencing or the characterization of individual miR activities via AGO1- versus AGO2-mediated regulation may also bear research values in the miR field, and these data, if available from future studies, would then significantly complement the accuracy and reliability of our model. Overall, the extensive simulations performed in this study identified and reasoned that overexpressing AGO1, TTP or antagonizing let-7 are effective strategies to suppress VEGF production in tumor, and that miR-15a antagonists alone, compared to other proposed strategies, could most potently enhance VEGF synthesis in simulated PAD conditions. Our model will advance the current understanding of how different miRs are regulated to affect angiogenesis when cells are actively adapting to hypoxia; it will also provide valuable insights into the future research in the pathophysiology of cancer and ischemic cardiovascular disease, including PAD, as well as the development of miR-based therapeutics that target other related pathways.

Sensitivity analysis reveals that the degree of HIF-1α stabilization is firmly controlled by the binding between O_2_ and hydroxylase enzymes (e.g. PHD2, FIH). The stronger O_2_ binds hydroxylase enzyme, the less HIF-1α is sustained both in normoxia and hypoxia. For tumor this change could be disadvantageous, since cells in hypoxia would fail to accumulate enough HIF-1α in order to trigger adequate pro-angiogenic adaptations. On the other side, inhibiting the interaction between O_2_ and hydroxylase enzyme increases HIF-1α signals and the expression of its targets, which potentially benefits patients with ischemic arterial disease [77]. We also pinpointed the molecule TTP, which destabilizes mRNA of multiple signaling molecules including its own, and we demonstrated *in silico* that it holds therapeutic value in tumor for its anti-angiogenic property [75, 78]. Although TTP overexpression seems more direct than miR-based therapies in terms of its mechanism to inhibit VEGF production, its mRNA-destabilizing activity is repressed upon phosphorylation by the mitogen-activated protein kinase [79, 80]. TTP is linked to miR as a direct target of miR-29a validated in cancer epithelial cells, which adds another layer of complexity to the delicate regulation of VEGF expression in hypoxic environments [81].

Speaking of potential model applications in the context of disease pathology, since the prominence of AGO1 and let-7 profiles in cancer has already been validated, the next step is to connect our model with miR features in vascular disease [21]. In the limited literature that studies miR dysregulation in PAD, circulating let-7 and miR-15a are shown to be downregulated in patients with PAD compared with healthy controls [82]. Interestingly, these miRs happened to be included in our model for their importance in the modulation of VEGF synthesis in hypoxia, and this motivates us to hypothesize the following mechanisms that could bridge the gap between miR data and PAD clinical symptoms, given the evidence that baseline VEGF levels in skeletal muscle of PAD patients were similar compared to healthy subjects [83]. In PAD patients, prolonged exposure to ischemia might turn on some epigenetic switch such as the LIN28/let-7 feedback loop that potently silences let-7 expression, so let-7 abundance is markedly reduced even in cell that has high HIF-1 level in response to hypoxic stimulus; as a result, AGO1 expression is less inhibited in hypoxia which leads to impaired VEGF desuppression and synthesis [84]. Therefore, the increase in tissue VEGF level might be insufficient to initiate an ischemic response to upregulate angiogenesis and re-establish perfusion in PAD patients. Since PAD prevalence increases significantly with age, another possible explanation about PAD-related miR feature looks at the loss-of-function of HIF-1 transcription factor in aging endothelial cells; nuclear import of HIF-1α is decreased in aging GMECs compared to young GMECs, and this severely blunts the induction of HIF-1 target genes including VEGF and let-7 in hypoxia [58, 85]. However, the mechanisms discussed above, including the model simulation of miR-based treatment for PAD, focused only on the regulation of pro-angiogenic cytokines, while pathophysiology of PAD is in fact affected by dysregulation of both pro- (e.g. VEGF) and anti-angiogenic factors (e.g. TSP-1, angiostatin) [86–88]. So far, efficacy of VEGF delivery strategies in PAD and CAD clinical trials is still below expectation, and systems biology studies that can quantitatively model multiscale cytokine interaction and angiogenesis will likely advance future drug designs for ischemic vascular disease [89]. Another limitation is that the model assumes a constantly low oxygen availability in ischemic tissues, which is more relevant to the clinical manifestation of patients with advanced-stage PAD since they tend to develop critical limb ischemia (CLI) and experience continuous leg pain, whereas early-stage PAD is often associated with intermittent claudication, suggesting that hypoxic exposure may be less prominent in this case [90–92].

Currently, the model describes only the dynamics of let-7 and miR-15a, but its setup allows future extensions to include more miRs and their target molecules. Ghosh et al. identified miR-424 as another HRM which targets CUL2 (cullin 2) in ECs; since CUL2 is essential in the assembly of the E3 ubiquitin ligase complex, miR-424 helps to stabilize HIF-1α and consequently increase the transcription of HIF-1 targets in hypoxia [93]. A few other hypoxia-responsive miRs, such as miR-155 and miR-210, have also been shown to negatively control HIF-1α stabilization in hypoxia by directly targeting its mRNA, and a basic but well-supported model describing the interactions between HIF-1α and miR-155 has been developed [32, 94]. In addition, members of miR-17/92 cluster are shown to target HIF-1α and TSP-1, and their biogenesis is repressed in hypoxia with a strong dependency on Dicer expression [95–97]. There is also evidence suggesting that AGO2, in response to hypoxia, is differentially regulated in tumor and smooth muscle cells, which in turn modulates the maturation of various miRs and influences downstream gene expressions [98, 99]. With all the experimental evidence, the model can be further enriched to depict a more comprehensive signal transduction network that starts with oxygen sensing, consists of diverse miR regulations, and ends at the secretion of pro- (e.g., VEGF) and anti-angiogenic (e.g., TSP-1) factors, with a better predicting power in terms of capturing both the general dynamics as a result of coordinated miR regulation and the specific profile changes of key intermediate species. So far, the model is formulated mostly based on validated knowledge in ECs, however, experimental evidence suggests that HIF-let-7-AGO-VEGF pathway is present in other cell/tissue types, including muscle and tumor, and it plays a fundamental role in the angiogenic adaptations of these cells in response to hypoxia [21]. This implies a possibility of making a model that predicts the proliferative and migratory behavior of individual cells based on cytokine signals (VEGF, TSP-1) released from all different cell types within a small population. In summary, our model is the first computational study that investigates miR control in hypoxia-induced angiogenesis and a pioneer in the field of miR mechanistic pathway models. To extend experiment-based mechanistic miR modeling to the next level, future computational studies could combine our work with state-of-the-art VEGF models and incorporate agent-based modeling techniques to simulate tissue-level proliferation and angiogenesis, within a bulk tissue from tumor or skeletal muscle, under different physiological conditions.

## Materials and Methods

### Formulation of reactions

We constructed the model which produces simulations for this study based on ordinary differential equations (ODE) with a total of 47 species, 91 kinetic parameters and 57 reactions (Fig 2). All reactions, with description and kinetic parameter values (S1 Table), and initial condition for each species (S2 Table) are available in the supporting information. Although the model does not focus on intracellular trafficking, for several species (e.g. HIF-1α) the model allows transportation and distinguishes them by cellular locations—in cytoplasm or in nucleus, since their core properties change along with the physical locations. Transcription activation and inhibition, along with Dicer processing are modeled as Hill-type or Michaelis-Menten kinetics, and we included turnover mechanisms for major species in order to capture more accurate long-term responses. 47 out of 57 reactions are modeled as first or second order biochemical reactions, and most interactions are based on experimental data from literature (S1 Table). All the data are put together into a computational model using MATLAB SimBiology toolbox (MathWorks, Natick, MA). Simulations are performed using the ode15s method, which is a stiff ODE solver provided in MATLAB. Since we are interested in the hypoxic response, the initial conditions of all species are their normoxic steady state levels which are obtained by a long enough simulation at 21% O_2_. For the simulation of different mRNA treatments, overexpression is equivalent to increasing its initial condition; silencing is modeled as the binding of siRNA with mRNA to form a compound in which mRNA is unusable. For miR treatments, overexpression by miR mimics increases the initial condition of the corresponding precursor miR, while silencing is reflected as the association of miR antagonist with miRISC to form a compound that cannot function. In the model validation section, to quantify the Western blot data from literature, we used ImageJ software (NIH) to perform densitometry analysis according to the blot analysis protocol. In the simulations that evaluate different treatments against PAD, the pathological setting of PAD is simplified and modeled as an impaired let-7 induction by hypoxia (value of kp21 in S1 Table is adjusted to 0.464 μM) according to the findings by Stather et al [74, 82].

Given the fact that time delays in processes such as transcription and translation are sometimes critical in determining the behaviors of certain biological systems, we showed in S9 Fig that a system built strictly with ODEs could still exhibit these necessary delays as a result of sufficient parameter tuning [100–102]. Although there has been little data that quantifies real-time transcription delays, our model predicts a delay in the range of 10 to 20 minutes for the transcription of VEGF and TTP (S9A and S9B Fig) which is very close to the experimental measurements by Ota et al [103]. S9D Fig compares the behavior of TTP in the original ODE model with the new behavior if TTP transcription/translation are modeled using delayed differential equations (DDEs) in MATLAB Simulink. Despite the large increase in its translation rate and a switch of modeling approach, the general time-course of TTP protein remains similar (S9D Fig). Therefore, we suggest that the important time delays in biological events are not overlooked by our ODE-based model given that we have done extensive parameter tuning and optimization, and the system’s core dynamics would be largely unaffected if the more explicit, DDE approach is taken instead of ODE.

### Model parameters and initial conditions

Due to the novelty of our model and the limited literature in miR modeling, we only take a few reaction parameters and initial conditions from published models on oxygen sensing [27]. To estimate the initial conditions for miRs, we compare published experimental data and assume that miRs are present in the order of 10^3^ to 10^4^ copies per cell in normoxia; the molar concentration is then computed using a 1 pL cell volume [104, 105]. Given the relatively low levels of miRs within the cell, some previous computational studies had employed the stochastic approach to model miR/protein regulatory networks [106, 107]. However, given the scope and complexity of our model, we choose to take the deterministic ODE approach after careful consideration. Mac Gabhann et al. observed that stochastic and deterministic simulations, respectively, of VEGF binding to its receptors would generate consistent results, although typical VEGF concentrations *in vivo* are in the picomolar range [108]. Similarly, Giamperi et al. found that the outcomes from stochastic and deterministic approaches in modeling a miR/protein toggle switch achieve a high degree of agreement [107]. The results of both studies corroborated our assumption that the deterministic approach we used captures the important average behavior of the system which would be of equal essence in the stochastic case.

The decay rates of mRNAs (1.2e-3 min^-1^), miRNAs (1e-4 min^-1^), proteins (2.5e-4 min^-1^), the rate of translation per mRNA (3 min^-1^), the normoxic level of various mRNAs (2.8e-5 μM) and proteins (0.08 μM) are estimated in a way that the values are within ±2 orders of magnitude compared to the global median value (normalized by 1 pL cell volume and shown in the brackets) found by large-scale quantification studies [109–111]. The rest of the reaction rates and initial conditions are either fitted or estimated based on empirical conjectures concluded by previous research (all the sources are summarized in S1 and S2 Tables). The optimization routine is carried out using the Levenberg-Marquardt algorithm within the *lsqnonlin* function available in MATLAB. Since EC time course data of miR signaling in hypoxia are very limited, we optimize the parameters by minimizing the sum of squared errors between normalized simulation profiles and two experimental datasets [21]. The optimized parameter set produces biologically justifiable simulations regarding the absolute concentrations of each species, and it also predicts time course VEGF expressions that fit well to other Western blot data performed in different cell types (see Results).

### Sensitivity analysis

Local sensitivity analysis was performed using the methods supplied in MATLAB SimBiology toolbox, which employs the “complex-step approximation” to compute time-dependent sensitivity of a species A with respect to a parameter X. For A, each sensitivity computed for a parameter was subjected to non-dimensionalization and integrated over the simulation time; then, the value was normalized to the sum of all integrals (each integral was calculated for a certain parameter), and the local sensitivity of A in hypoxia was visualized in a pie chart. To evaluate the impact of adjusting local parameters on the relative difference between species expressions in normoxia and in hypoxia, a script was written in MATLAB to repeatedly run the following events in order: vary kinetic parameters, simulate the model to obtain new steady states in normoxia, set the new steady states as model initial conditions for hypoxia and simulate the model in hypoxia to calculate relative expressions.

## Supporting Information

S1 Table Reaction descriptions, reaction rates and kinetic parameters. (PDF)

S2 Table Model differential equations and species initial conditions. (PDF)

S1 Fig A rapid decline in free form AGO1 mRNA lead to the decline of total intracellular AGO1. (PDF)

S2 Fig Experimental time course profiles of let-7 and model simulations of intracellular let-7, VEGF in hypoxia. (PDF)

S3 Fig AGO1 binds let-7 and prevents it from degradation. (PDF)

S4 Fig AGO1 overexpression reduces VEGF production (experiment and simulation). (PDF)

S5 Fig Effect of anti-angiogenic strategies in extreme hypoxia. (PDF)

S6 Fig Comparison between different treatments for PAD *in silico*. (PDF)

S7 Fig Additional sensitivity analysis of selected parameters affiliated with pathway signature molecules. (PDF)

S8 Fig Increased binding of O_2_ with HIF hydroxylases reduces the effect of HIF stabilization in both normoxia and hypoxia. (PDF)

S9 Fig Time delays in the modeling of biological processes and comparison between ODE and DDE approaches. (PDF)

S1 File Full supporting information for the study. The file includes S1 and S2 Tables, which contain the reactions, descriptions, parameters and initial conditions used in the model. A glossary of abbreviations used in this study, and S1–S9 Figs are also included which show additional related results.
(PDF)
