# Supplementary material for: Computational Model of MicroRNA Control of HIF-VEGF Pathway: Insights into the Pathophysiology of Ischemic Vascular Disease and Cancer
Source: PLoS Comput Biol. 2015 Nov 20;11(11):e1004612. doi: 10.1371/journal.pcbi.1004612 (PMC4654485; doi:10.1371/journal.pcbi.1004612)
Supplement: S1 Table — (PDF) [file pcbi.1004612.s001.pdf]

S1_Table: Reaction descriptions, reaction rates, kinetic parameters

No.	Reaction description	Reaction Rate	Ref.
	**Oxygen Sensing Module**		
v1	TTP represses HIF1α translation	$vm1 * (1 - \frac{TTP\hat{}n1}{(kp1\hat{}n1+TTP\hat{}n1)})$, vm1=0.012 μM/min, n1=4, kp1=0.552 μM	[1]
v2	HIF1α translocation	$kf2 * HIF1\alpha - kr2 * HIF1\alpha_N$, kf2=0.005 min^{-1}, kr2=0.018 min^{-1}	[2]
v3	FIH complex binds HIF1α	$kf3 * HIF1\alpha * [FIH\text{–}O_2\text{–}Fe\text{–}DG] - kr3 * [HIF1\alpha\text{–}FIH\ complex]$, kf3=0.13 $\mu M^{-1}min^{-1}$, kr3=1 min^{-1}	[3]
v4	O_2 binds FIH-DG-Fe	$kf4 * O_2 * [FIH\text{–}Fe\text{–}DG] - kr4 * [FIH\text{–}O_2\text{–}Fe\text{–}DG]$, kf4=0.215 $\mu M^{-1}min^{-1}$, kr4=10.6 min^{-1}	[3]
v5	DG binds FIH-Fe	$kf5 * [FIH\text{–}Fe] * DG - kr5 * [FIH\text{–}Fe\text{–}DG]$, kf5=0.23 $\mu M^{-1}min^{-1}$, kr5=7.4 min^{-1}	[3]
v6	Fe binds FIH	$kf6 * Fe * FIH - kr6 * [FIH/FE]$, kf6=4 $\mu M^{-1}min^{-1}$, kr6=10 min^{-1}	[3]
v7	PHD2 complex binds HIF1α	$kf7 * [PHD2\text{–}O_2\text{–}Fe\text{–}DG] * HIF1\alpha - kr7 * [HIF1\alpha\text{–}PHD\ complex]$, kf7=0.11 $\mu M^{-1}min^{-1}$, kr7=0.7 min^{-1}	[2]
v8	O_2 binds PHD2-Fe-DG	$kf8 * O_2 * [PHD2\text{–}Fe\text{–}DG] - kr8 * [PHD2\text{–}O_2\text{–}Fe\text{–}DG]$, kf8=0.043 $\mu M^{-1}min^{-1}$, kr8=10.8 min^{-1}	[2]
v9	DG binds PHD2-Fe	$kf9 * [PHD2\text{–}Fe] * DG - kr9 * [PHD2\text{–}DG]$, kf9=0.18 $\mu M^{-1}min^{-1}$, kr9=10.8 min^{-1}	[2]
v10	Fe binds PHD2	$kf10 * PHD2 * Fe - kr10 * [PHD2\text{–}Fe]$, kf10=18 $\mu M^{-1}min^{-1}$, kr10=36 min^{-1}	[2]
v11	$CoCl_2$ inhibits HIF1α hydroxylation by FIH	$kf11 * [HIF1\alpha\text{–}FIH\ complex] * (1 - CoCl_2/(CoCl_2 + kp11))$, kf11=0.34 min^{-1}, kp11=60 μM	[3]
v12	$CoCl_2$ inhibits HIF1α hydroxylation by PHD2	$kf12 * [HIF1\alpha\text{–}PHD\ complex] * (1 - CoCl_2/(CoCl_2 + kp12))$, kf12=0.44 min^{-1}, kp12=90 μM	[3]
v13	VHL recognizes hydroxylated HIF1α	$kf13 * VHL * [HIF1\alpha/OH] - kr13 * [HIF1\alpha/OH\text{–}VHL]$, kf13=42 $\mu M^{-1}min^{-1}$, kr13=1.3 min^{-1}	[2]
v14	VHL-motivated degradation of HIF1α	$kf14 * [HIF1\alpha/OH\text{–}VHL]$, kf14=1 min^{-1}	[2]
v15	HIF1α dimerizes with HIF1β in the nucleus	$kf15 * HIF1\beta_N * HIF1\alpha_N - kr15 * [HIF1\text{–}dimer_N]$, kf15=0.006 $\mu M^{-1}min^{-1}$, kr15=0.03 min^{-1}	[2]
v16	TTP degradation	$kf16 * [TTP]$, kf16=0.002 min^{-1}	Fitted
v17	TTP translation	$kf17 * [mTTp]$, kf17=0.11 min^{-1}	Fitted
	**HIF-dependent Transcription Module**		
v18	TTP mRNA degradation	$kf18 * [mTTP]$, kf18=0.004 min^{-1}	Fitted
v19	HIF-1 complex activates TTP transcription	$vm19 * \frac{[HIF1\text{–}dimer_N]\hat{}n19}{(kp19\hat{}n19+[HIF1\text{–}dimer_N]\hat{}n19)}$, vm19=5e-5 μM/min, n19=2, kp19=0.03 μM	Fitted
v20	HIF-1 complex activates VEGF transcription	$vm20 * (0.03 + \frac{[HIF1\text{–}dimer_N]\hat{}n20}{kp20\hat{}n20+[HIF1\text{–}dimer_N]\hat{}n20})$, vm20=7.85e-7 μM/min, kp20=0.0583 μM, n20=2	[4]

No.	Reaction Description	Reaction Rate	Ref.
v21	HIF-1 complex activates let-7 transcription	$vm21 * \frac{[HIF1\text{-}dimer_N]^\wedge n21}{kp21^\wedge n21+[HIF1\text{-}dimer_N]^\wedge n21}$, vm21=6.01e-4 μM/min, kp21=0.0451 μM, n21=3	[5]
v22	TTP represses VEGF translation	$kf22 * mVEGFA * \left(1 - \frac{TTP^\wedge n22}{kp22^\wedge n22+TTP^\wedge n22}\right)$, kf22= 0.75 min^{-1}, kp22=0.678 μM, n22=2	[6]
v23	VEGF degradation	$kf23 * VEGFA$, kf23=0.0016 min^{-1}	Fitted
v24	VEGF mRNA degradation	$kf24 * mVEGFA$, kf24=0.0083 min^{-1}	Fitted
	**VEGF Repression by MiR-15a Module**		
v25	miR-15a transcription	1.7e-5 μM/min	[7]
v26	Pri-miR-15a nuclear export	$kf26 * [Pri\text{-}miR\text{-}15a_N]$, kf26=0.005 min^{-1}	[7]
v27	Pre-miR-15a degradation	$kf27 * [Pre\text{-}miR\text{-}15a]$, kf27=0.001 min^{-1}	Fitted
v28	Pre-miR-15a Dicer cleavage	$vm28 * Dicer * \frac{[Pre\text{-}miR\text{-}15a]}{kp28+[Pre\text{-}miR\text{-}15a]}$, vm28=0.001 min^{-1}, kp28=0.25 μM	[8]
v29	miR-15a degradation	$kf29 * [miR\text{-}15a]$, kf29=8e-4 min^{-1}	Fitted
v30	Formation of miR-15a RISC by miR-15a and AGO1	$kf30 * [miR\text{-}15a] * AGO1 - kr30 * [miR\text{-}15a\ RISC]$, kf30=0.4 $\mu M^{-1} min^{-1}$, kr30=1 min^{-1}	Fitted
v31	miR-15a RISC binds and inhibits VEGFA mRNA	$kf31 * mVEGFA * [miR\text{-}15a\ RISC] - kr31 * [miR\text{-}15a\ RISC\text{-}mVEGFA]$, kf31=8 $\mu M^{-1} min^{-1}$, kr31=0.14 min^{-1}	[9]
v32	Repressed mVEGFA localizes to p-body	$kf32 * [miR\text{-}15a\ RISC\text{-}mVEGFA]$, kf32=1.2 min^{-1}	[10]
v33	p-body mVEGFA degradation	$kf33 * [mVEGFA/p\text{-}body]$, kf33=2e-5 min^{-1}	Fitted
v34	p-body mVEGFA escape	$kf34 * [mVEGFA/p\text{-}body]$, kf34=0.001 min^{-1}	[11]
	**Let-7 Biogenesis and Targeting Module**		
v35	Pre-let-7 degradation	$kf35 * [Pre\text{-}let\text{-}7]$, kf35=0.01 min^{-1}	Fitted
v36	Let-7 RISC regulates pri-let-7 processing and export	$[Pri\text{-}let\text{-}7_N] * vm36 * \frac{[let\text{-}7\ RISC]^\wedge n36}{kp36^\wedge n36+[let\text{-}7\ RISC]^\wedge n36}$, vm36=5 min^{-1}, kp36=0.0349 μM, n36=2	[12]
v37	Let-7 Dicer cleavage	$vm37 * Dicer * \frac{[Pre\text{-}let\text{-}7]}{kp37+[Pre\text{-}let\text{-}7]}$, vm37=0.01 min^{-1}, kp37=0.03 μM	[13]
v38	Let-7 degradation	$kf38 * [let\text{-}7]$, kf38=0.008 min^{-1}	Fitted
v39	AGO1 mRNA degradation	$kf39 * mAGO1$, kf39=1e-4 min^{-1}	Fitted
v40	mAGO1 production	6.01e-7 μM/min	Fitted
v41	AGO1 translation	$kf41 * mAGO1$, kf41=1.275 min^{-1}	Fitted
v42	Formation of let-7 RISC by let-7 and AGO1	$kf42 * [let\text{-}7] * AGO1 - kr42 * [let\text{-}7\ RISC]$, kf42=1 $\mu M^{-1} min^{-1}$, kr42=0.07 min^{-1}	[5]
v43	Let-7 RISC binds and inhibits AGO1 mRNA	$kf43 * mAGO1 * [let\text{-}7\ RISC] - kr43 * [let\text{-}7\ RISC\text{-}mAGO1]$, kf43=8 $\mu M^{-1} min^{-1}$, kr43=0.15 min^{-1}	[5]
v44	Repressed mAGO1 localizes to p-body	$kf44 * [let\text{-}7\ RISC\text{-}mAGO1]$, kf44=0.3 min^{-1}	[10]
v45	p-body mAGO1 degradation	$kf45 * [mAGO1/p\text{-}body]$, kf45=2.63e-5 min^{-1}	Fitted
v46	p-body mAGO1 escape	$kf46 * [mAGO1/p\text{-}body]$, kf46=0.00103 min^{-1}	[11]
v47	mDicer production	2.4e-5 μM/min	Fitted

No.	Reaction Description	Reaction Rate	Ref.
v48	mDicer degradation	$kf48 * mDicer$, kf48=0.008 min^{-1}	Fitted
v49	Let-7 RISC binds and inhibits Dicer mRNA	$kf49 * mDicer * [let\text{-}7\ RISC] - kr49 * [let\text{-}7\ RISC\text{-}mDicer]$, kf49=6.9 $\mu M^{-1}min^{-1}$, kr49=0.09 min^{-1}	[13]
v50	Repressed mDicer localizes to p-body	$kf50 * [let\text{-}7\ RISC\text{-}mDicer]$, kf50=1.8 min^{-1}	[10]
v51	p-body mDicer degradation	$kf51 * [mDicer/p\text{-}body]$, kf51=2e-5 min^{-1}	Fitted
v52	p-body mDicer escape	$kf52 * [mDicer/p\text{-}body]$, kf52=0.002 min^{-1}	[11]
v53	Dicer translation	$kf53 * mDicer$, kf53=0.5 min^{-1}	Fitted
v54	Dicer degradation	$kf54 * dicer$, kf54=0.0014 min^{-1}	Fitted
v55	AGO1 degradation	$kf55 * AGO1$, kf55=0.0021 min^{-1}	Fitted
v56	Antagonizing miR (let-7, miR-15a) using LNA	$kf56 * LNA * [miR\ RISC] - kr56 * [miR\ RISC\text{-}LNA]$, kf56=20 $min^{-1}\mu M^{-1}$, kr56=0.02 min^{-1}	[14]
v57	Silencing mRNAs using siRNA	$kf57 * siRNA * mRNA - kr57 * [mRNA\text{-}siRNA]$, kf57=20 $min^{-1}\mu M^{-1}$, kr57=0.02 min^{-1}	Fitted

S1_Table. Reaction descriptions, reaction rates and kinetic parameters. Reactions are formulated based on experimental evidence in the literature denoted with corresponding reference numbers. Reaction rates v1-v57 here match with the numbers in the model scheme provided in the article. $Species_N$ represents that the species is in the nucleus, other species, assumed in protein or miR form, are in the cytoplasm; mSpecies represents the mRNA of the species. Parameters kf3-kf10, kr3-kr10 are estimated based on reaction rates in published models [15]. Other parameters are fitted or estimated according to large scale global quantification studies [16-19].

Abbreviations used in the model components

Abbreviations	Species full name
TTP	Tristetraprolin
HIF-1α	Hypoxia-inducible factor 1 alpha subunit
HIF-1β	Hypoxia-inducible factor 1 beta subunit
FIH-1	Factor inhibiting HIF-1
O_2	Oxygen
Fe	Iron
DG	2-oxoglutarate
PHD2	Prolyl hydroxylase domain-containing protein 2

$CoCl_2$	Cobalt chloride
VHL	Von Hippel-Lindau E3 ubiquitin protein ligase
VEGF	Vascular endothelial growth factor
AGO1	Argonaute 1
LNA	Locked nucleic acid
RISC	RNA-induced silencing complex

References

1. Kim TW, Yim S, Choi BJ, Jang Y, Lee JJ, Sohn BH, et al. Tristetraprolin regulates the stability of HIF-1alpha mRNA during prolonged hypoxia. Biochemical and biophysical research communications. 2010;391(1):963-8. doi: 10.1016/j.bbrc.2009.11.174. PubMed PMID: 19962963.
2. Ke Q, Costa M. Hypoxia-inducible factor-1 (HIF-1). Molecular pharmacology. 2006;70(5):1469-80. doi: 10.1124/mol.106.027029. PubMed PMID: 16887934.
3. Lando D, Peet DJ, Gorman JJ, Whelan DA, Whitelaw ML, Bruick RK. FIH-1 is an asparaginyl hydroxylase enzyme that regulates the transcriptional activity of hypoxia-inducible factor. Genes & development. 2002;16(12):1466-71. doi: 10.1101/gad.991402. PubMed PMID: 12080085; PubMed Central PMCID: PMC186346.
4. Forsythe JA, Jiang BH, Iyer NV, Agani F, Leung SW, Koos RD, et al. Activation of vascular endothelial growth factor gene transcription by hypoxia-inducible factor 1. Molecular and cellular biology. 1996;16(9):4604-13. PubMed PMID: 8756616; PubMed Central PMCID: PMC231459.
5. Chen Z, Lai TC, Jan YH, Lin FM, Wang WC, Xiao H, et al. Hypoxia-responsive miRNAs target argonaute 1 to promote angiogenesis. The Journal of clinical investigation. 2013;123(3):1057-67. doi: 10.1172/JCI65344. PubMed PMID: 23426184; PubMed Central PMCID: PMC3582133.
6. Ross CR, Brennan-Laun SE, Wilson GM. Tristetraprolin: roles in cancer and senescence. Ageing research reviews. 2012;11(4):473-84. doi: 10.1016/j.arr.2012.02.005. PubMed PMID: 22387927; PubMed Central PMCID: PMC3376680.
7. Bartel DP. MicroRNAs: genomics, biogenesis, mechanism, and function. Cell. 2004;116(2):281-97. PubMed PMID: 14744438.
8. Hebert SS, Papadopoulou AS, Smith P, Galas MC, Planel E, Silahtaroglu AN, et al. Genetic ablation of Dicer in adult forebrain neurons results in abnormal tau hyperphosphorylation and neurodegeneration. Human molecular genetics. 2010;19(20):3959-69. doi: 10.1093/hmg/ddq311. PubMed PMID: 20660113.
9. Yin KJ, Olsen K, Hamblin M, Zhang J, Schwendeman SP, Chen YE. Vascular endothelial cell-specific microRNA-15a inhibits angiogenesis in hindlimb ischemia. The Journal of biological chemistry. 2012;287(32):27055-64. doi: 10.1074/jbc.M112.364414. PubMed PMID: 22692216; PubMed Central PMCID: PMC3411046.
10. Eulalio A, Behm-Ansmant I, Schweizer D, Izaurralde E. P-body formation is a consequence, not the cause, of RNA-mediated gene silencing. Molecular and cellular biology. 2007;27(11):3970-81. doi: 10.1128/MCB.00128-07. PubMed PMID: 17403906; PubMed Central PMCID: PMC1900022.
11. Parker R, Sheth U. P bodies and the control of mRNA translation and degradation. Molecular cell. 2007;25(5):635-46. doi: 10.1016/j.molcel.2007.02.011. PubMed PMID: 17349952.
12. Zisoulis DG, Kai ZS, Chang RK, Pasquinelli AE. Autoregulation of microRNA biogenesis by let-7 and Argonaute. Nature. 2012;486(7404):541-4. doi: 10.1038/nature11134. PubMed PMID: 22722835; PubMed Central PMCID: PMC3387326.

13. Tokumaru S, Suzuki M, Yamada H, Nagino M, Takahashi T. let-7 regulates Dicer expression and constitutes a negative feedback loop. Carcinogenesis. 2008;29(11):2073-7. doi: 10.1093/carcin/bgn187. PubMed PMID: 18700235.
14. Stenvang J, Petri A, Lindow M, Obad S, Kauppinen S. Inhibition of microRNA function by antimiR oligonucleotides. Silence. 2012;3(1):1. doi: 10.1186/1758-907X-3-1. PubMed PMID: 22230293; PubMed Central PMCID: PMC3306207.
15. Qutub AA, Popel AS. A computational model of intracellular oxygen sensing by hypoxia-inducible factor HIF1 alpha. Journal of cell science. 2006;119(Pt 16):3467-80. doi: 10.1242/jcs.03087. PubMed PMID: 16899821; PubMed Central PMCID: PMC2129128.
16. Bissels U, Wild S, Tomiuk S, Holste A, Hafner M, Tuschl T, et al. Absolute quantification of microRNAs by using a universal reference. Rna. 2009;15(12):2375-84. doi: 10.1261/rna.1754109. PubMed PMID: 19861428; PubMed Central PMCID: PMC2779673.
17. Schwanhausser B, Busse D, Li N, Dittmar G, Schuchhardt J, Wolf J, et al. Corrigendum: Global quantification of mammalian gene expression control. Nature. 2013;495(7439):126-7. doi: 10.1038/nature11848. PubMed PMID: 23407496.
18. Yang E, van Nimwegen E, Zavolan M, Rajewsky N, Schroeder M, Magnasco M, et al. Decay rates of human mRNAs: correlation with functional characteristics and sequence attributes. Genome research. 2003;13(8):1863-72. doi: 10.1101/gr.1272403. PubMed PMID: 12902380; PubMed Central PMCID: PMC403777.
19. Gantier MP, McCoy CE, Rusinova I, Saulep D, Wang D, Xu D, et al. Analysis of microRNA turnover in mammalian cells following Dicer1 ablation. Nucleic acids research. 2011;39(13):5692-703. doi: 10.1093/nar/gkr148. PubMed PMID: 21447562; PubMed Central PMCID: PMC3141258.
