# Supplementary material for: Computational Model of MicroRNA Control of HIF-VEGF Pathway: Insights into the Pathophysiology of Ischemic Vascular Disease and Cancer
Source: PLoS Comput Biol. 2015 Nov 20;11(11):e1004612. doi: 10.1371/journal.pcbi.1004612 (PMC4654485; doi:10.1371/journal.pcbi.1004612)
Supplement: S2 Table — (PDF) [file pcbi.1004612.s002.pdf]

S2_Table: Differential equations and species initial conditions

Species	Differential Equations ($\frac{d[Species]}{dt}$)	Species Initial Condition (μM)
	**Oxygen Sensing Module**	
HIF1α	v1-v2-v3-v7	0.173
HIF1α-FIH complex	v3-v12	0.0183
HIF1α-PHD complex	v7-v13	0.0131
FIH-O_2-Fe-DG	v4+v12-v3	1.09
O_2	-v4-v8	Nx(21% O_2):209, Hx (2% O_2):19.9
FIH-DG-Fe	v5-v4	0.257
DG	-v5-v9	999
FIH-Fe	v6-v5	8.28e-3
Fe	-v6-v10	49.6
FIH	-v6	4.17e-4
$CoCl_2$	Constant	0
HIF1α/OH	v11+v12-v13	5.39e-4
VHL	v14-v13	1.22
HIF1α/OH-VHL	v13-v14	0.0120
PHD2-O_2-Fe-DG	v12+v8-v7	0.788
PHD2-Fe-DG	v9-v8	0.947
PHD2-Fe	v10-v9	0.0568
PHD2	-v10	2.29e-3
HIF1α_N	v2-v15	0.0480
HIF1β_N	-v15	0.883
TTP	v17-v16	0.0509
	**HIF-dependent Transcription Module**	
mTTP	v19-v18	9.35e-4
HIF1-dimer$_N$	v15	8.48e-3
mVEGFA	v20-v24-v31+v34	4.49e-6
VEGFA	v22-v23	2.09e-3
Pri-let-7$_N$	v21-v36	6.16e-5
	**VEGF Repression by MiR-15a Module**	
Pri-miR-15a$_N$	v25-v26	3.40e-3
Pre-miR-15a	v26-v27-v28	3.33e-3
Dicer	v53-v54	1.04
miR-15a	v28-v29-v30	0.0171
AGO1	v41-v55-v42-v30	0.581
miR-15a RISC	v30-v31+v32	3.97e-3
miR-15a RISC-mVEGFA	v31-v32	1.06e-7
mVEGFA/p-body	v32-v33-v34	1.25e-4
	**Let-7 Biogenesis and Targeting Module**	
Pre-let-7	v36-v35-v37	1.11e-5
Let-7	v37-v38-v42	4.79e-4
Let-7 RISC	v42-v49-v43+v44+v50	3.98e-3
mAGO1/p-body	v44-v45-v46	0.0192

Species	Differential Equations ($\frac{d[Species]}{dt}$)	Species Initial Condition (μM)
mAGO1	v40-v39-v43+v46	9.52e-4
Let-7 RISC-mAGO1	v43-v44	6.74e-5
mDicer	v47-v48-v49+v52	2.91e-3
Let-7 RISC-mDicer	v49-v50	4.22e-5
mDicer/p-body	v50-v51-v52	0.0376
LNA, miR RISC	-v56	0
miR RISC-LNA	v56	0
siRNA, mRNA	-v57	0
mRNA-siRNA	v57	0

S2_Table. Model differential equations and species initial conditions. Initial conditions here also refer to the steady-state (normoxia) concentration of each species. The different O_2 initial condition in the simulation corresponds to different O_2 abundance. PHD2, FIH, Fe, O_2, DG, HIF1α initial conditions are estimated based on the measurements made by Tuckerman et al [1]. $CoCl_2$ initial condition is changed to 200 μM to mimic hypoxia in a normoxic O_2 environment, and in all other simulations the level of $CoCl_2$ is zero [2]. To maintain a moderate complexity, the model assumes that transcription factors or enzymes in Hill-type (Michaelis-Menten) reactions are unconsumed, and mRNAs are unconsumed in translation.

References

1. Tuckerman JR, Zhao Y, Hewitson KS, Tian YM, Pugh CW, Ratcliffe PJ, et al. Determination and comparison of specific activity of the HIF-prolyl hydroxylases. FEBS letters. 2004;576(1-2):145-50. doi: 10.1016/j.febslet.2004.09.005. PubMed PMID: 15474027.
2. Liu Q, Xu Z, Mao S, Chen W, Zeng R, Zhou S, et al. Effect of hypoxia on hypoxia inducible factor-1alpha, insulin-like growth factor I and vascular endothelial growth factor expression in hepatocellular carcinoma HepG2 cells. Oncology letters. 2015;9(3):1142-8. doi: 10.3892/ol.2015.2879. PubMed PMID: 25663870; PubMed Central PMCID: PMC4315007.
