# Supplementary material for: Computational Model of MicroRNA Control of HIF-VEGF Pathway: Insights into the Pathophysiology of Ischemic Vascular Disease and Cancer
Source: PLoS Comput Biol. 2015 Nov 20;11(11):e1004612. doi: 10.1371/journal.pcbi.1004612 (PMC4654485; doi:10.1371/journal.pcbi.1004612)
Supplement: S1 Fig — (PDF) [file pcbi.1004612.s003.pdf]

S1_Fig

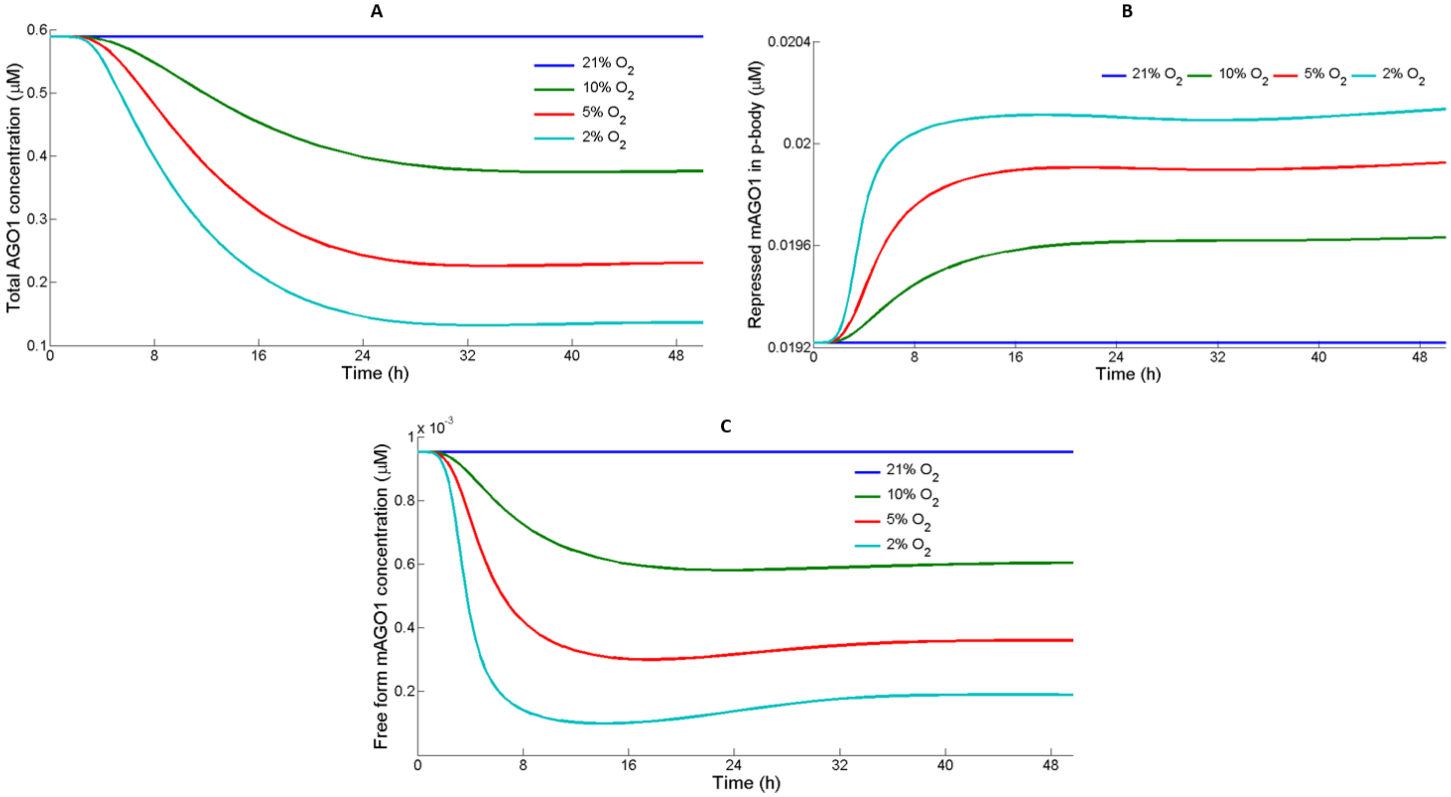

S1_Fig. A rapid decline in free form AGO1 mRNA lead to the decline of total intracellular AGO1. As oxygen level drops, (A) Total AGO1 level declines; (B) more mAGO1 is targeted by let-7 RISC and directed to p-body for temporary storage, leading to a decrease in the (C) free form mAGO1 that are ready to be translated. The lower the oxygen availability, the quicker the change in mAGO1 takes place, the steeper the initial drop in total AGO1.
