# Supplementary material for: Computational Model of MicroRNA Control of HIF-VEGF Pathway: Insights into the Pathophysiology of Ischemic Vascular Disease and Cancer
Source: PLoS Comput Biol. 2015 Nov 20;11(11):e1004612. doi: 10.1371/journal.pcbi.1004612 (PMC4654485; doi:10.1371/journal.pcbi.1004612)
Supplement: S2 Fig — (PDF) [file pcbi.1004612.s004.pdf]

S2_Fig

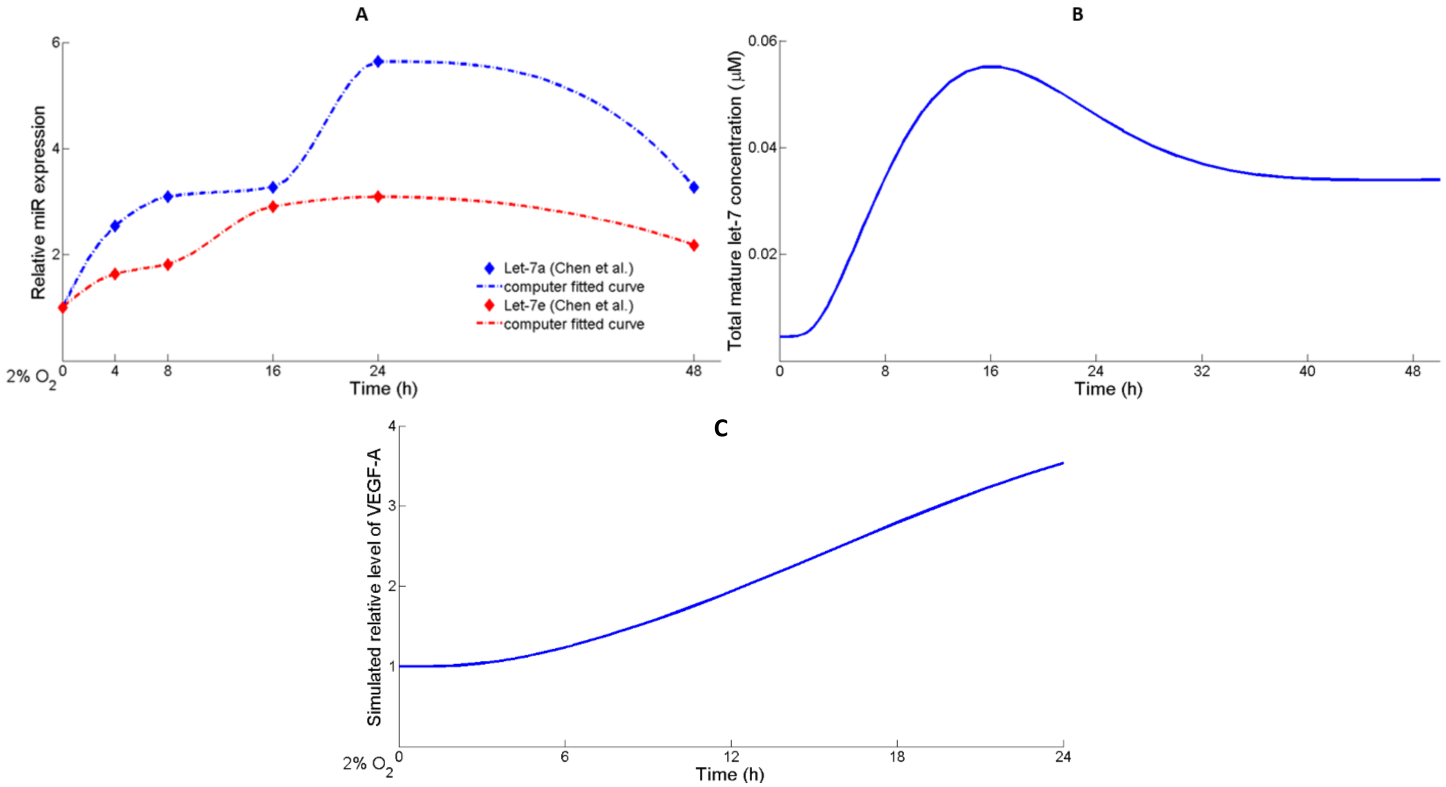

S2_Fig. Experimental time course profiles of let-7 and model simulations of intracellular let-7, VEGF in hypoxia. (A) Experimental quantification of total mature let-7s induced in 2% oxygen in endothelial cell by Chen et al. at discrete time points; values (symbols) are normalized to initial expression and dashed curves are computer interpolated trajectories [1]. (B) In agreement with the trend shown in the experimental data, model simulation predicts an initial steep rise and a subsequent drop in total let-7 level due in 2% oxygen. (C) Model simulation of intracellular VEGF predicts a 3.5 fold induction (relative to the normoxic baseline level) in response to a 24-hour exposure to 2% O_2.

References

1. Chen Z, Lai TC, Jan YH, Lin FM, Wang WC, Xiao H, et al. Hypoxia-responsive miRNAs target argonaute 1 to promote angiogenesis. The Journal of clinical investigation. 2013;123(3):1057-67. doi: 10.1172/JCI65344. PubMed PMID: 23426184; PubMed Central PMCID: PMC3582133.
