# Supplementary material for: Computational Model of MicroRNA Control of HIF-VEGF Pathway: Insights into the Pathophysiology of Ischemic Vascular Disease and Cancer
Source: PLoS Comput Biol. 2015 Nov 20;11(11):e1004612. doi: 10.1371/journal.pcbi.1004612 (PMC4654485; doi:10.1371/journal.pcbi.1004612)
Supplement: S3 Fig — (PDF) [file pcbi.1004612.s005.pdf]

S3_Fig

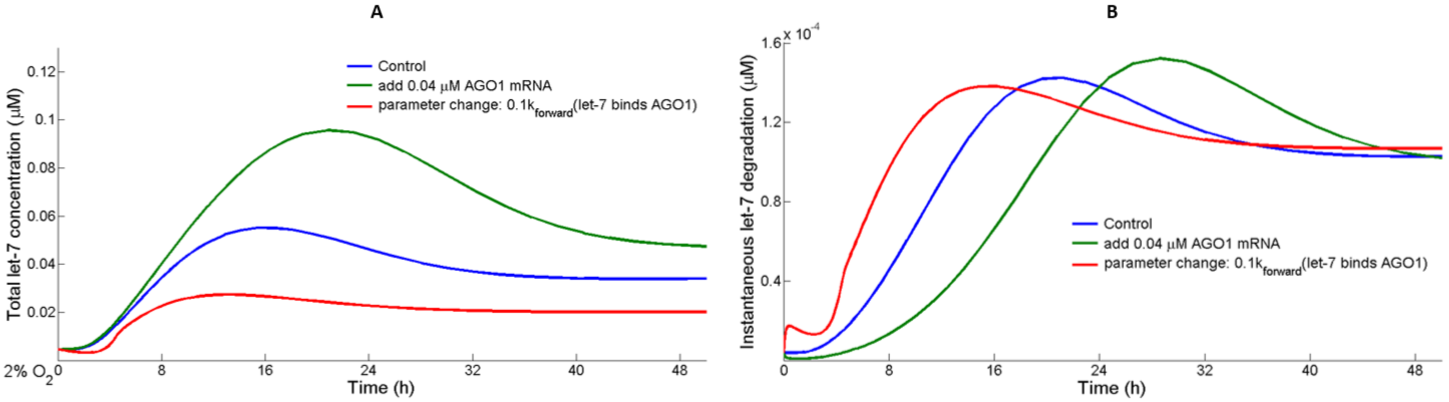

S3_Fig. AGO1 binds let-7 and prevents it from degradation. (A) Enforced overexpression of AGO1 binds more free form let-7 and stabilizes the total let-7 level in hypoxia, while a weak association between AGO1 and let-7 causes more let-7 degradation. (B) In the curves showing instantaneous degradation of let-7 in hypoxia, the case of weak AGO1/let-7 binding has larger let-7 degradation in the beginning; the case of AGO1 overexpression has much smaller let-7 degradation. Since the steady state levels of free let-7 are comparable in all three cases, the obvious differences in total let-7 levels are contributed by the additional let-7 stored in let-7/AGO1 complexes.
