# Supplementary material for: Computational Model of MicroRNA Control of HIF-VEGF Pathway: Insights into the Pathophysiology of Ischemic Vascular Disease and Cancer
Source: PLoS Comput Biol. 2015 Nov 20;11(11):e1004612. doi: 10.1371/journal.pcbi.1004612 (PMC4654485; doi:10.1371/journal.pcbi.1004612)
Supplement: S4 Fig — (PDF) [file pcbi.1004612.s006.pdf]

S4_Fig

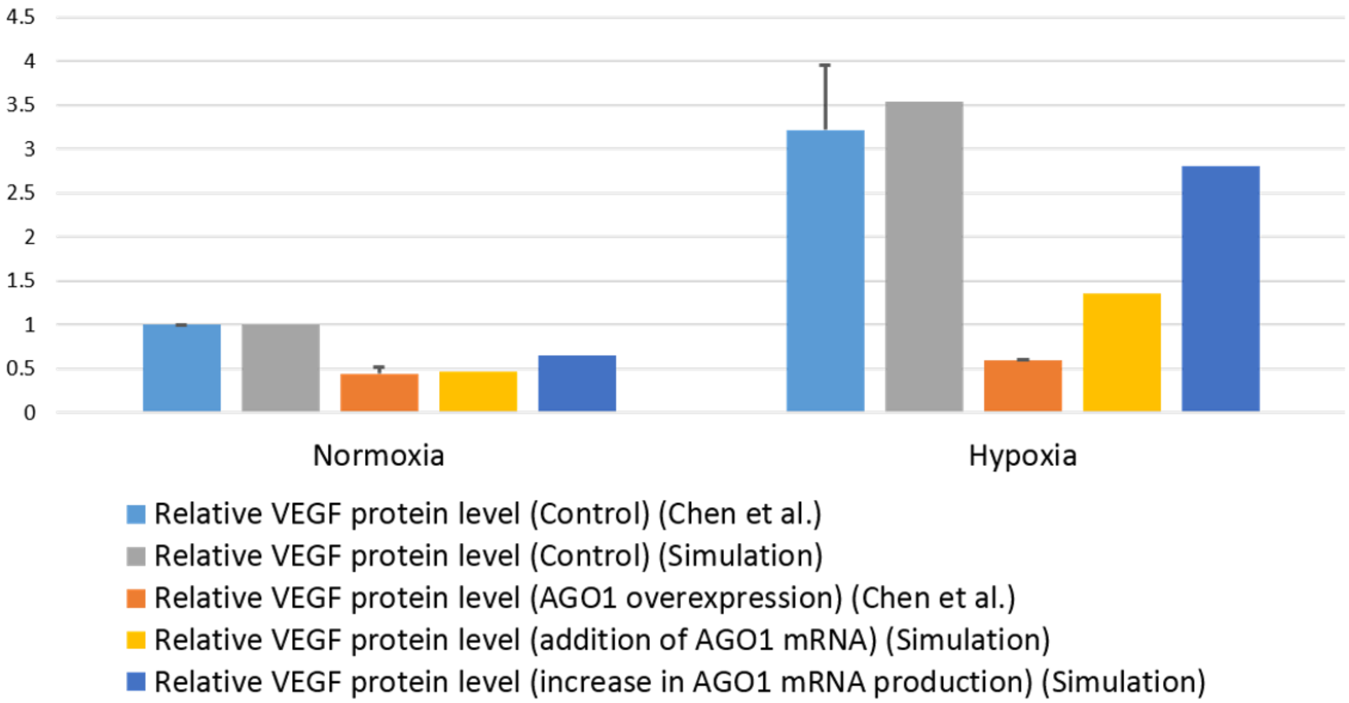

S4_Fig. AGO1 overexpression reduces VEGF production. Experiments by Chen et al. verified that AGO1 overexpression decreases VEGF protein level in HUVECs in normoxia and in hypoxia. We numerically quantified the Western blot data provided in literature [1]. Standard deviations were calculated and relative expressions were normalized to the normoxic VEGF protein level with no additional AGO1 introduced. Model simulations of AGO1 overexpression by two different approaches, increase the initial AGO1 mRNA by 0.08 μM or increase the AGO1 mRNA production rate by 50 fold, result in reductions of intracellular VEGF protein levels that are in qualitative agreement with the experiments. Because of the lack of relevant information in the experimental protocol, we can only approximate the values of the simulation parameters that were used to mimic the effect of *in vitro* AGO1 overexpression, and simulated VEGF protein levels at 24 hours were measured and compared to experimental Western blot quantifications.

References

1. Chen Z, Lai TC, Jan YH, Lin FM, Wang WC, Xiao H, et al. Hypoxia-responsive miRNAs target argonaute 1 to promote angiogenesis. The Journal of clinical investigation. 2013;123(3):1057-67. doi: 10.1172/JCI65344. PubMed PMID: 23426184; PubMed Central PMCID: PMC3582133.
