# Supplementary material for: Computational Model of MicroRNA Control of HIF-VEGF Pathway: Insights into the Pathophysiology of Ischemic Vascular Disease and Cancer
Source: PLoS Comput Biol. 2015 Nov 20;11(11):e1004612. doi: 10.1371/journal.pcbi.1004612 (PMC4654485; doi:10.1371/journal.pcbi.1004612)
Supplement: S5 Fig — (PDF) [file pcbi.1004612.s007.pdf]

# S5_Fig

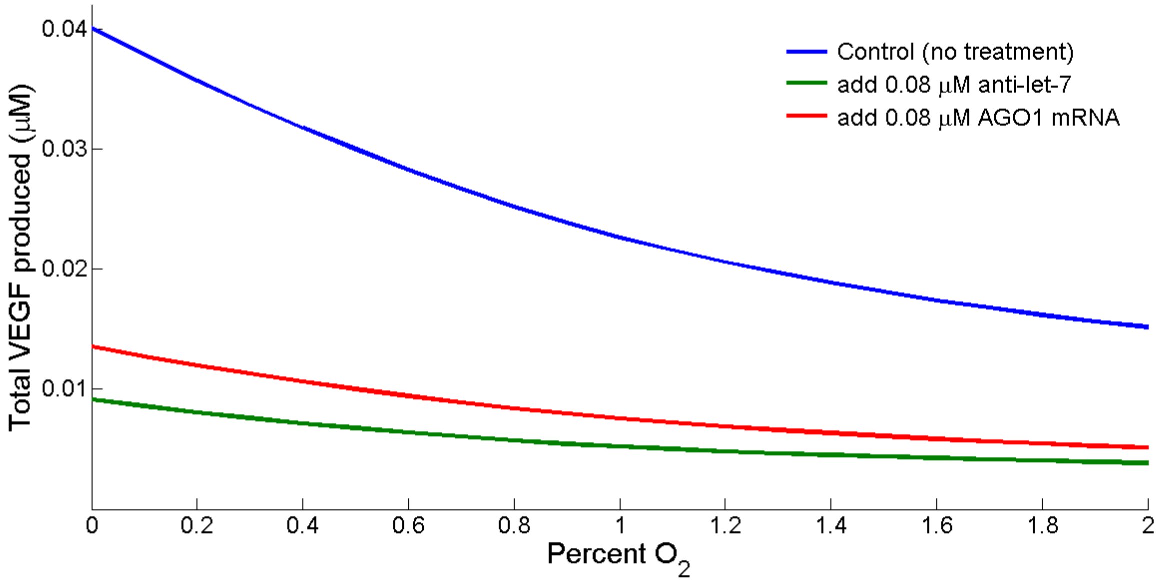


**S5_Fig. Effect of anti-angiogenic strategies in extreme hypoxia.** In extremely low $O_2$ concentrations (0-2%) that mimic tumor microenvironment, simulations show that using let-7 antagonists (green) or overexpressing AGO1 (red) significantly reduces the total VEGF synthesized during a 24-hour span by nearly four fold, compared to the untreated situation (blue).
