# Supplementary material for: Computational Model of MicroRNA Control of HIF-VEGF Pathway: Insights into the Pathophysiology of Ischemic Vascular Disease and Cancer
Source: PLoS Comput Biol. 2015 Nov 20;11(11):e1004612. doi: 10.1371/journal.pcbi.1004612 (PMC4654485; doi:10.1371/journal.pcbi.1004612)
Supplement: S6 Fig — (PDF) [file pcbi.1004612.s008.pdf]

S6_Fig

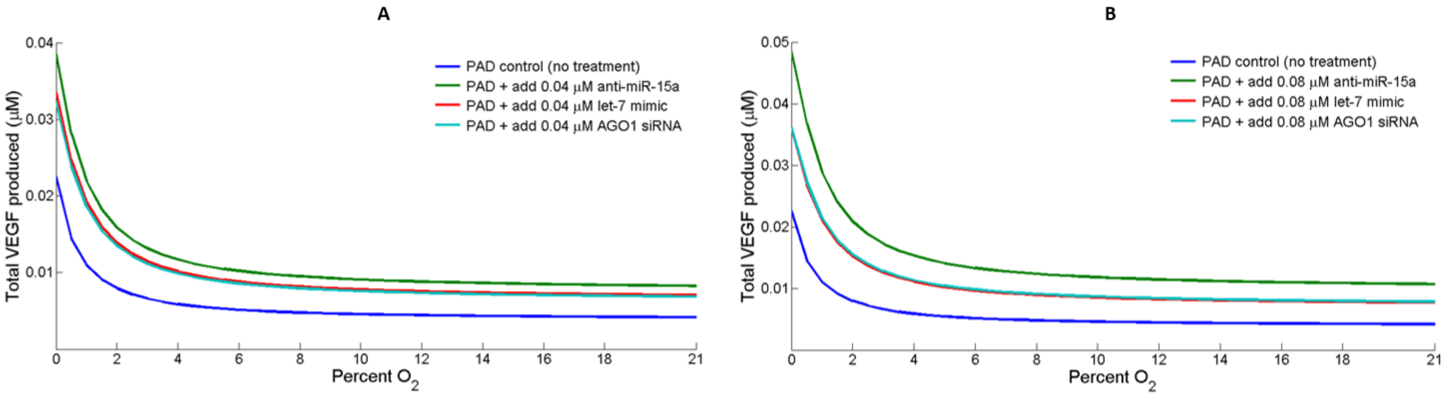

S6_Fig. Comparison between different treatments for PAD *in silico*. (A) Measurement of total VEGF produced in a 24-hour span in response to 0.04 µM of each treatment, and (B) 0.08 µM of each treatment. Since antagonizing miR-15a immediately releases VEGF mRNA from the miR-15a RISC while the other two strategies exert their effect indirectly, miR-15a antagonist is more effective in stimulating VEGF synthesis compared to let-7 mimic or AGO1 siRNA, at the same dose level in a simulated PAD environment.
