# Supplementary material for: Computational Model of MicroRNA Control of HIF-VEGF Pathway: Insights into the Pathophysiology of Ischemic Vascular Disease and Cancer
Source: PLoS Comput Biol. 2015 Nov 20;11(11):e1004612. doi: 10.1371/journal.pcbi.1004612 (PMC4654485; doi:10.1371/journal.pcbi.1004612)
Supplement: S7 Fig — (PDF) [file pcbi.1004612.s009.pdf]

S7_Fig

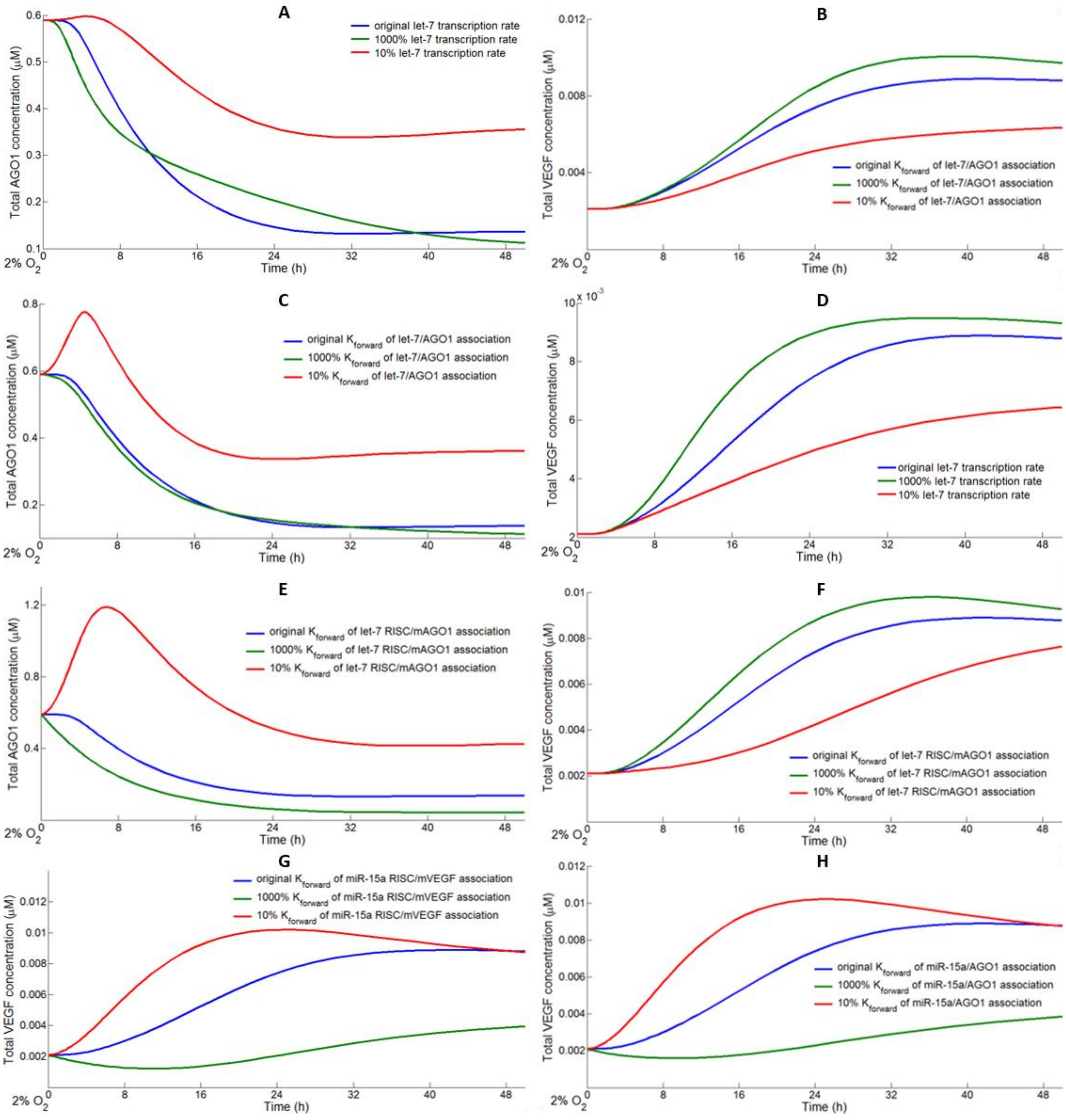
A
original let-7 transcription rate
1000% let-7 transcription rate
10% let-7 transcription rate
Total AGO1 concentration (μM)
2% O2
Time (h)
B
original Kforward of let-7/AGO1 association
1000% Kforward of let-7/AGO1 association
10% Kforward of let-7/AGO1 association
Total VEGF concentration (μM)
2% O2
Time (h)
C
original Kforward of let-7/AGO1 association
1000% Kforward of let-7/AGO1 association
10% Kforward of let-7/AGO1 association
Total AGO1 concentration (μM)
2% O2
Time (h)
D
x 10-3
original let-7 transcription rate
1000% let-7 transcription rate
10% let-7 transcription rate
Total VEGF concentration (μM)
2% O2
Time (h)
E
original Kforward of let-7 RISC/mAGO1 association
1000% Kforward of let-7 RISC/mAGO1 association
10% Kforward of let-7 RISC/mAGO1 association
Total AGO1 concentration (μM)
2% O2
Time (h)
F
original Kforward of let-7 RISC/mAGO1 association
1000% Kforward of let-7 RISC/mAGO1 association
10% Kforward of let-7 RISC/mAGO1 association
Total VEGF concentration (μM)
2% O2
Time (h)
G
original Kforward of miR-15a RISC/mVEGF association
1000% Kforward of miR-15a RISC/mVEGF association
10% Kforward of miR-15a RISC/mVEGF association
Total VEGF concentration (μM)
2% O2
Time (h)
H
original Kforward of miR-15a/AGO1 association
1000% Kforward of miR-15a/AGO1 association
10% Kforward of miR-15a/AGO1 association
Total VEGF concentration (μM)
2% O2
Time (h)

S7_Fig. Additional sensitivity analysis of selected reaction rates affiliated with major species in the pathway. Modular sensitivity analysis presented in the article identified several reaction parameters that can most significantly influence the behaviors of the model, so here we performed additional analysis specific to these parameters. Varying the rate of let-7 transcription by $\pm$10 fold result in different magnitudes of (A) AGO1 downregulation and (B) VEGF induction. Disrupting the association between let-7 and AGO1, rather than enforcing the binding, has a more profound impact on (C) AGO1 dynamics and (D) leads to a siginificant temporal delay in the induction of VEGF, while decreasing the forward binding rate of let-7 RISC with AGO1 mRNA causes a more significant change to (E) AGO1 profile and it further delayes (F) VEGF synthesis. Lastly, either varying the rate of VEGF targeting by miR-15a RISC or the rate of miR-15a/AGO1 association has very little impact on the time course AGO1 dynamics (data not shown), and both parameters, when varied by $\pm$ 10 fold respectively, result in comparable VEGF expression curves (G-H). The initial rise in AGO1 dynamics observed in (C and E) is due to the fact that all three simulations are based on the same initial conditions, which allows more straightforward comparisons; still, the continuing decrease in AGO1 expression is the dominant behavior during the simulation span. These results reflect the intrinsic robustness of the model, which is demonstrated by these analysis to exhibit highly consistent dynamics/patterns with only minor quantitative changes upon parameter variations. Therefore, the major conclusions drawn based on the overall qualitative behavior of the key species in the model (e.g. AGO1, VEGF), in spite of the fact uncertainty exists in the parameter space for a reasonable range, would potentially remain unchanged.
