# Supplementary material for: Computational Model of MicroRNA Control of HIF-VEGF Pathway: Insights into the Pathophysiology of Ischemic Vascular Disease and Cancer
Source: PLoS Comput Biol. 2015 Nov 20;11(11):e1004612. doi: 10.1371/journal.pcbi.1004612 (PMC4654485; doi:10.1371/journal.pcbi.1004612)
Supplement: S8 Fig — (PDF) [file pcbi.1004612.s010.pdf]

S8_Fig

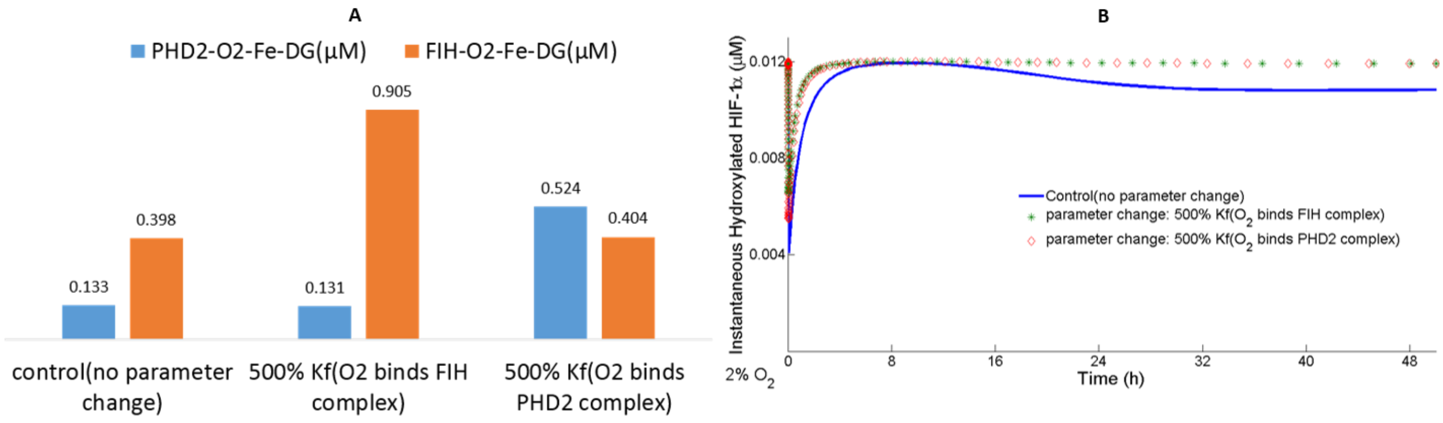

S8_Fig. Increased binding of O_2 with HIF hydroxylases reduces the effect of HIF stabilization in both normoxia and hypoxia. Here we selectively increase the forward binding rate of O_2 with hydroxylase 5 fold; in response to this, (A) in hypoxia (2% oxygen), steady state total compound formation of O_2 with hydroxylases increases, which (B) speeds up the instantaneous hydroxylation of HIF-1α during the simulation compared to the control case (blue curve). The hydroxylation rate indicated by green asterisks (increased FIH binding) does not differ significantly from the rate indicated by red diamonds (increased PHD2 binding), but these two cases have different HIF-1α initial conditions so the relative fold changes computed are not identical.
