# Supplementary material for: Computational Model of MicroRNA Control of HIF-VEGF Pathway: Insights into the Pathophysiology of Ischemic Vascular Disease and Cancer
Source: PLoS Comput Biol. 2015 Nov 20;11(11):e1004612. doi: 10.1371/journal.pcbi.1004612 (PMC4654485; doi:10.1371/journal.pcbi.1004612)
Supplement: S9 Fig — (PDF) [file pcbi.1004612.s011.pdf]

S9_Fig

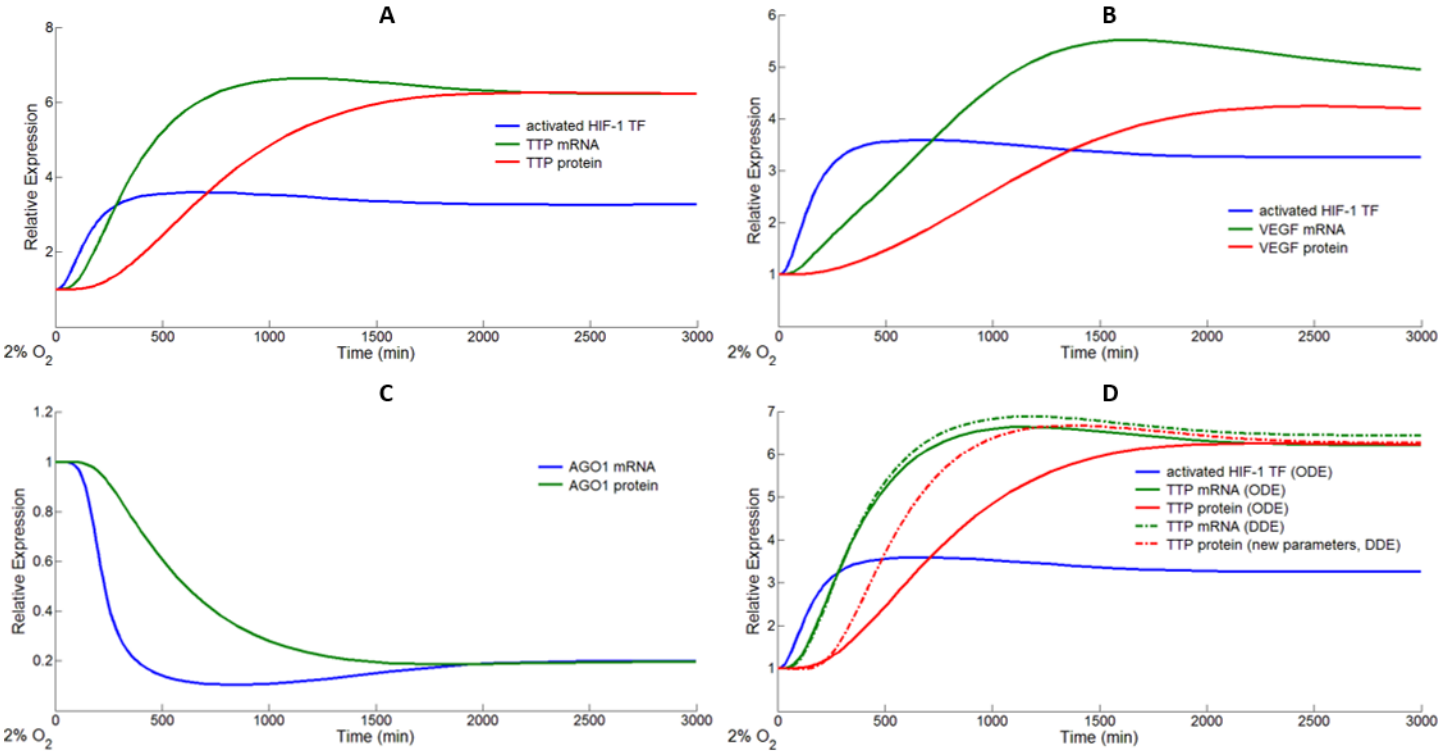

S9_Fig. Inherited time delays in the model and comparisons between ODE and DDE implementations. The computational model implemented in ordinary differential equations (ODEs) has inherited time delays in processes such as transcription and translation of (A) TTP, (B) VEGF, and (C) AGO1. (D) Re-calculating a subpart of the model, in this case the synthesis of TTP, by delayed differential equations (DDE) while increasing TTP tranlsation (v17) and TTP protein degradation (v16) rate by 4.5-fold produces time curves that are simliar to results obtained in the original ODE implementation. The faster production/degradation rates tested in the DDE approach give rise to a small difference in the TTP protein curve (an early overshoot), but its influence on the original model behavior is insignificant since the TTP protein steady states, which determine the final HIF-1α levels, are very similar in both approaches. This suggests that our model, an ODE system with tuned reaction parameters, can reproduce the effect of time delays in real biological systems, and that its core behaviors would not alter qualitatively if we implement the model in DDE with a new set of fitted parameters.
