# Supplementary material for: Computational Model of MicroRNA Control of HIF-VEGF Pathway: Insights into the Pathophysiology of Ischemic Vascular Disease and Cancer
Source: PLoS Comput Biol. 2015 Nov 20;11(11):e1004612. doi: 10.1371/journal.pcbi.1004612 (PMC4654485; doi:10.1371/journal.pcbi.1004612)
Supplement: S1 File — The file includes S1 and S2 Tables, which contain the reactions, descriptions, parameters and initial conditions used in the model. A glossary of abbreviations used in this study, and S1–S9 Figs are also included which show additional related results. (PDF) [file pcbi.1004612.s012.pdf]

Full Supporting Information

S1_Table: Reaction descriptions, reaction rates, kinetic parameters

No.	Reaction description	Reaction Rate	Ref.
	**Oxygen Sensing Module**		
v1	TTP represses HIF1α translation	$vm1 * (1 - \frac{TTP\string^n1}{(kp1\string^n1+TTP\string^n1)})$, vm1=0.012 μM/min, n1=4, kp1=0.552 μM	[1]
v2	HIF1α translocation	$kf2 * HIF1\alpha\ -\ kr2 * HIF1\alpha_N$, kf2=0.005 min^{-1}, kr2=0.018 min^{-1}	[2]
v3	FIH complex binds HIF1α	$kf3 * HIF1\alpha * [FIH{-}O_2{-}Fe{-}DG]\ -\ kr3 * [HIF1\alpha{-}FIH\ complex]$, kf3=0.13 $\mu M^{-1}min^{-1}$, kr3=1 min^{-1}	[3]
v4	O_2 binds FIH-DG-Fe	$kf4 * O_2 * [FIH{-}Fe{-}DG]\ -\ kr4 * [FIH{-}O_2{-}Fe{-}DG]$, kf4=0.215 $\mu M^{-1}min^{-1}$, kr4=10.6 min^{-1}	[3]
v5	DG binds FIH-Fe	$kf5 * [FIH{-}Fe] * DG\ -\ kr5 * [FIH{-}Fe{-}DG]$, kf5=0.23 $\mu M^{-1}min^{-1}$, kr5=7.4 min^{-1}	[3]
v6	Fe binds FIH	$kf6 * Fe * FIH\ -\ kr6 * [FIH/FE]$, kf6=4 $\mu M^{-1}min^{-1}$, kr6=10 min^{-1}	[3]
v7	PHD2 complex binds HIF1α	$kf7 * [PHD2{-}O_2{-}Fe{-}DG] * HIF1\alpha\ -\ kr7 * [HIF1\alpha{-}PHD\ complex]$, kf7=0.11 $\mu M^{-1}min^{-1}$, kr7=0.7 min^{-1}	[2]
v8	O_2 binds PHD2-Fe-DG	$kf8 * O_2 * [PHD2{-}Fe{-}DG] -\ kr8 * [PHD2{-}O_2{-}Fe{-}DG]$, kf8=0.043 $\mu M^{-1}min^{-1}$, kr8=10.8 min^{-1}	[2]
v9	DG binds PHD2-Fe	$kf9 * [PHD2{-}Fe] * DG\ -\ kr9 * [PHD2{-}DG]$, kf9=0.18 $\mu M^{-1}min^{-1}$, kr9=10.8 min^{-1}	[2]
v10	Fe binds PHD2	$kf10 * PHD2 * Fe - kr10 * [PHD2{-}Fe]$, kf10=18 $\mu M^{-1}min^{-1}$, kr10=36 min^{-1}	[2]
v11	$CoCl_2$ inhibits HIF1α hydroxylation by FIH	$kf11 * [HIF1\alpha{-}FIH\ complex] * (1 - CoCl_2/(CoCl_2 + kp11))$, kf11=0.34 min^{-1}, kp11=60 μM	[3]
v12	$CoCl_2$ inhibits HIF1α hydroxylation by PHD2	$kf12 * [HIF1\alpha{-}PHD\ complex] * (1 - CoCl_2/(CoCl_2 + kp12))$, kf12=0.44 min^{-1}, kp12=90 μM	[3]
v13	VHL recognizes hydroxylated HIF1α	$kf13 * VHL * [HIF1\alpha/OH]\ -\ kr13 * [HIF1\alpha/OH{-}VHL]$, kf13=42 $\mu M^{-1}min^{-1}$, kr13=1.3 min^{-1}	[2]
v14	VHL-motivated degradation of HIF1α	$kf14 * [HIF1\alpha/OH{-}VHL]$, kf14=1 min^{-1}	[2]
v15	HIF1α dimerizes with HIF1β in the nucleus	$kf15 * HIF1\beta_N * HIF1\alpha_N\ -\ kr15 * [HIF1{-}dimer_N]$, kf15=0.006 $\mu M^{-1}min^{-1}$, kr15=0.03 min^{-1}	[2]
v16	TTP degradation	$kf16 * [TTP]$, kf16=0.002 min^{-1}	Fitted
v17	TTP translation	$kf17 * [mTTp]$, kf17=0.11 min^{-1}	Fitted
	**HIF-dependent Transcription Module**		
v18	TTP mRNA degradation	$kf18 * [mTTP]$, kf18=0.004 min^{-1}	Fitted
v19	HIF-1 complex activates TTP transcription	$vm19 * \frac{[HIF1{-}dimer_N]\string^n19}{(kp19\string^n19+[HIF1{-}dimer_N]\string^n19)}$, vm19=5e-5 μM/min, n19=2, kp19=0.03 μM	Fitted
v20	HIF-1 complex activates VEGF transcription	$vm20 * (0.03 + \frac{[HIF1{-}dimer_N]\string^n20}{kp20\string^n20+[HIF1{-}dimer_N]\string^n20})$, vm20=7.85e-7 μM/min, kp20=0.0583 μM, n20=2	[4]

No.	Reaction Description	Reaction Rate	Ref.
v21	HIF-1 complex activates let-7 transcription	$vm21 * \frac{[HIF1\text{-}dimer_N]^\wedge n21}{kp21^\wedge n21+[HIF1\text{-}dimer_N]^\wedge n21}$, vm21=6.01e-4 μM/min, kp21=0.0451 μM, n21=3	[5]
v22	TTP represses VEGF translation	$kf22 * mVEGFA * \left(1 - \frac{TTP^\wedge n22}{kp22^\wedge n22+TTP^\wedge n22}\right)$, kf22= 0.75 min^{-1}, kp22=0.678 μM, n22=2	[6]
v23	VEGF degradation	$kf23 * VEGFA$, kf23=0.0016 min^{-1}	Fitted
v24	VEGF mRNA degradation	$kf24 * mVEGFA$, kf24=0.0083 min^{-1}	Fitted
	**VEGF Repression by MiR-15a Module**		
v25	miR-15a transcription	1.7e-5 μM/min	[7]
v26	Pri-miR-15a nuclear export	$kf26 * [Pri\text{-} miR\text{-} 15a_N]$, kf26=0.005 min^{-1}	[7]
v27	Pre-miR-15a degradation	$kf27 * [Pre\text{-} miR\text{-} 15a]$, kf27=0.001 min^{-1}	Fitted
v28	Pre-miR-15a Dicer cleavage	$vm28 * Dicer * \frac{[Pre\text{-}miR\text{-}15a]}{kp28+[Pre\text{-}miR\text{-}15a]}$, vm28=0.001 min^{-1}, kp28=0.25 μM	[8]
v29	miR-15a degradation	$kf29 * [miR\text{-} 15a]$, kf29=8e-4 min^{-1}	Fitted
v30	Formation of miR-15a RISC by miR-15a and AGO1	$kf30 * [miR\text{-} 15a] * AGO1 - kr30 * [miR\text{-} 15a\ RISC]$, kf30=0.4 $\mu M^{-1}min^{-1}$, kr30=1 min^{-1}	Fitted
v31	miR-15a RISC binds and inhibits VEGFA mRNA	$kf31 * mVEGFA * [miR\text{-} 15a\ RISC] - kr31 * [miR\text{-} 15a\ RISC\text{-} mVEGFA]$, kf31=8 $\mu M^{-1}min^{-1}$, kr31=0.14 min^{-1}	[9]
v32	Repressed mVEGFA localizes to p-body	$kf32 * [miR\text{-} 15a\ RISC\text{-} mVEGFA]$, kf32=1.2 min^{-1}	[10]
v33	p-body mVEGFA degradation	$kf33 * [mVEGFA/p\text{-} body]$, kf33=2e-5 min^{-1}	Fitted
v34	p-body mVEGFA escape	$kf34 * [mVEGFA/p\text{-} body]$, kf34=0.001 min^{-1}	[11]
	**Let-7 Biogenesis and Targeting Module**		
v35	Pre-let-7 degradation	$kf35 * [Pre\text{-} let\text{-} 7]$, kf35=0.01 min^{-1}	Fitted
v36	Let-7 RISC regulates pri-let-7 processing and export	$[Pri\text{-} let\text{-} 7_N] * vm36 * \frac{[let\text{-}7\ RISC]^\wedge n36}{kp36^\wedge n36+[let\text{-}7\ RISC]^\wedge n36}$, vm36=5 min^{-1}, kp36=0.0349 μM, n36=2	[12]
v37	Let-7 Dicer cleavage	$vm37 * Dicer * \frac{[Pre\text{-}let\text{-}7]}{kp37+[Pre\text{-}let\text{-}7]}$, vm37=0.01 min^{-1}, kp37=0.03 μM	[13]
v38	Let-7 degradation	$kf38 * [let\text{-} 7]$, kf38=0.008 min^{-1}	Fitted
v39	AGO1 mRNA degradation	$kf39 * mAGO1$, kf39=1e-4 min^{-1}	Fitted
v40	mAGO1 production	6.01e-7 μM/min	Fitted
v41	AGO1 translation	$kf41 * mAGO1$, kf41=1.275 min^{-1}	Fitted
v42	Formation of let-7 RISC by let-7 and AGO1	$kf42 * [let\text{-} 7] * AGO1 - kr42 * [let\text{-} 7\ RISC]$, kf42=1 $\mu M^{-1}min^{-1}$, kr42=0.07 min^{-1}	[5]
v43	Let-7 RISC binds and inhibits AGO1 mRNA	$kf43 * mAGO1 * [let\text{-} 7\ RISC] - kr43 * [let\text{-} 7\ RISC\text{-} mAGO1]$, kf43=8 $\mu M^{-1}min^{-1}$, kr43=0.15 min^{-1}	[5]
v44	Repressed mAGO1 localizes to p-body	$kf44 * [let\text{-} 7\ RISC\text{-} mAGO1]$, kf44=0.3 min^{-1}	[10]
v45	p-body mAGO1 degradation	$kf45 * [mAGO1/p\text{-} body]$, kf45=2.63e-5 min^{-1}	Fitted
v46	p-body mAGO1 escape	$kf46 * [mAGO1/p\text{-} body]$, kf46=0.00103 min^{-1}	[11]
v47	mDicer production	2.4e-5 μM/min	Fitted

No.	Reaction Description	Reaction Rate	Ref.
v48	mDicer degradation	kf48 ∗ mDicer, kf48=0.008 min^{-1}	Fitted
v49	Let-7 RISC binds and inhibits Dicer mRNA	kf49 ∗ mDicer ∗ [let− 7 RISC] − kr49 ∗ [let− 7 RISC− mDicer], kf49=6.9 $\mu M^{-1} min^{-1}$, kr49=0.09 min^{-1}	[13]
v50	Repressed mDicer localizes to p-body	kf50 ∗ [let− 7 RISC− mDicer], kf50=1.8 min^{-1}	[10]
v51	p-body mDicer degradation	kf51 ∗ [mDicer/p− body], kf51=2e-5 min^{-1}	Fitted
v52	p-body mDicer escape	kf52 ∗ [mDicer/p− body], kf52=0.002 min^{-1}	[11]
v53	Dicer translation	kf53 ∗ mDicer, kf53=0.5 min^{-1}	Fitted
v54	Dicer degradation	kf54 ∗ dicer, kf54=0.0014 min^{-1}	Fitted
v55	AGO1 degradation	kf55 ∗ AGO1, kf55=0.0021 min^{-1}	Fitted
v56	Antagonizing miR (let-7, miR-15a) using LNA	kf56 ∗ LNA ∗ [miR RISC] − kr56 ∗ [miR RISC− LNA], kf56=20 $min^{-1} \mu M^{-1}$, kr56=0.02 min^{-1}	[14]
v57	Silencing mRNAs using siRNA	kf57 ∗ siRNA ∗ mRNA − kr57 ∗ [mRNA− siRNA], kf57=20 $min^{-1} \mu M^{-1}$, kr57=0.02 min^{-1}	Fitted

S1_Table. Reaction descriptions, reaction rates and kinetic parameters. Reactions are formulated based on experimental evidence in the literature denoted with corresponding reference numbers. Reaction rates v1-v57 here match with the numbers in the model scheme provided in the article. $Species_N$ represents that the species is in the nucleus, other species, assumed in protein or miR form, are in the cytoplasm; mSpecies represents the mRNA of the species. Parameters kf3-kf10, kr3-kr10 are estimated based on reaction rates in published models [15]. Other parameters are fitted or estimated according to large scale global quantification studies [16-19].

Abbreviations used in the model components

Abbreviations	Species full name
TTP	Tristetraprolin
HIF-1α	Hypoxia-inducible factor 1 alpha subunit
HIF-1β	Hypoxia-inducible factor 1 beta subunit
FIH-1	Factor inhibiting HIF-1
O_2	Oxygen
Fe	Iron
DG	2-oxoglutarate
PHD2	Prolyl hydroxylase domain-containing protein 2
$CoCl_2$	Cobalt chloride
VHL	Von Hippel-Lindau E3 ubiquitin protein ligase
VEGF	Vascular endothelial growth factor
AGO1	Argonaute 1
LNA	Locked nucleic acid
RISC	RNA-induced silencing complex

S2_Table: Differential equations and species initial conditions

Species	Differential Equations ($\frac{d[\text{Species}]}{dt}$)	Species Initial Condition (μM)
	**Oxygen Sensing Module**	
HIF1α	v1-v2-v3-v7	0.173
HIF1α-FIH complex	v3-v12	0.0183
HIF1α-PHD complex	v7-v13	0.0131
FIH-O_2-Fe-DG	v4+v12-v3	1.09
O_2	-v4-v8	Nx(21% O_2):209, Hx (2% O_2):19.9
FIH-DG-Fe	v5-v4	0.257
DG	-v5-v9	999
FIH-Fe	v6-v5	8.28e-3
Fe	-v6-v10	49.6
FIH	-v6	4.17e-4
$CoCl_2$	Constant	0
HIF1α/OH	v11+v12-v13	5.39e-4
VHL	v14-v13	1.22
HIF1α/OH-VHL	v13-v14	0.0120
PHD2-O_2-Fe-DG	v12+v8-v7	0.788
PHD2-Fe-DG	v9-v8	0.947
PHD2-Fe	v10-v9	0.0568
PHD2	-v10	2.29e-3
HIF1α_N	v2-v15	0.0480
HIF1β_N	-v15	0.883
TTP	v17-v16	0.0509
	**HIF-dependent Transcription Module**	
mTTP	v19-v18	9.35e-4
HIF1-dimer$_N$	v15	8.48e-3
mVEGFA	v20-v24-v31+v34	4.49e-6
VEGFA	v22-v23	2.09e-3
Pri-let-7$_N$	v21-v36	6.16e-5
	**VEGF Repression by MiR-15a Module**	
Pri-miR-15a$_N$	v25-v26	3.40e-3
Pre-miR-15a	v26-v27-v28	3.33e-3
Dicer	v53-v54	1.04
miR-15a	v28-v29-v30	0.0171
AGO1	v41-v55-v42-v30	0.581
miR-15a RISC	v30-v31+v32	3.97e-3
miR-15a RISC-mVEGFA	v31-v32	1.06e-7
mVEGFA/p-body	v32-v33-v34	1.25e-4
	**Let-7 Biogenesis and Targeting Module**	
Pre-let-7	v36-v35-v37	1.11e-5
Let-7	v37-v38-v42	4.79e-4
Let-7 RISC	v42-v49-v43+v44+v50	3.98e-3
mAGO1/p-body	v44-v45-v46	0.0192

Species	Differential Equations ($\frac{\mathbf{d[Species]}}{\mathbf{dt}}$)	Species Initial Condition (μM)
mAGO1	v40-v39-v43+v46	9.52e-4
Let-7 RISC-mAGO1	v43-v44	6.74e-5
mDicer	v47-v48-v49+v52	2.91e-3
Let-7 RISC-mDicer	v49-v50	4.22e-5
mDicer/p-body	v50-v51-v52	0.0376
LNA, miR RISC	-v56	0
miR RISC-LNA	v56	0
siRNA, mRNA	-v57	0
mRNA-siRNA	v57	0

S2_Table. Model differential equations and species initial conditions. Initial conditions here also refer to the steady-state (normoxia) concentration of each species. The different O_2 initial condition in the simulation corresponds to different O_2 abundance. PHD2, FIH, Fe, O_2, DG, HIF1α initial conditions are estimated based on the measurements made by Tuckerman et al [20]. $CoCl_2$ initial condition is changed to 200 μM to mimic hypoxia in a normoxic O_2 environment, and in all other simulations the level of $CoCl_2$ is zero [21]. To maintain a moderate complexity, the model assumes that transcription factors or enzymes in Hill-type (Michaelis-Menten) reactions are unconsumed, and mRNAs are unconsumed in translation.

S1_Fig

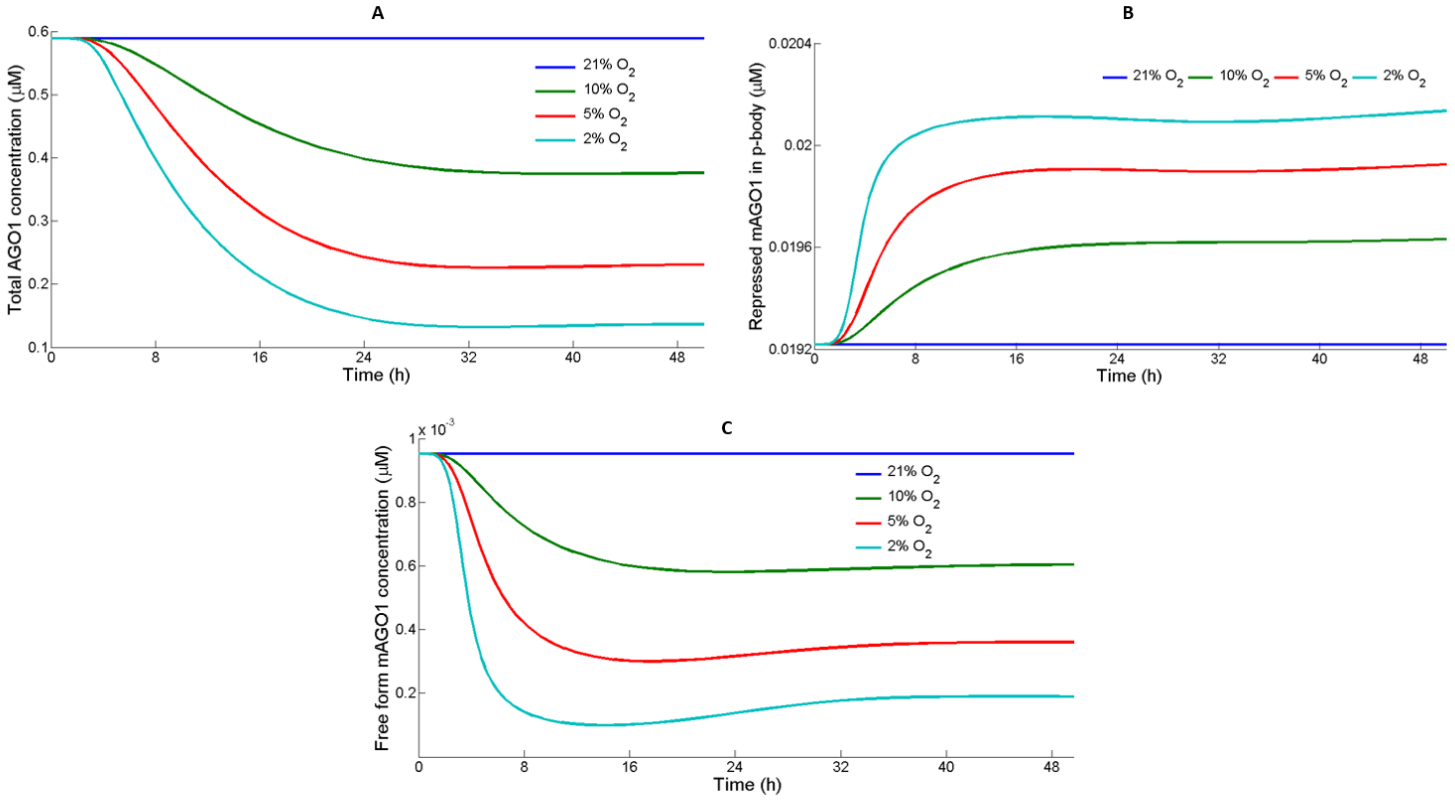

S1_Fig. A rapid decline in free form AGO1 mRNA lead to the decline of total intracellular AGO1. As oxygen level drops, (A) Total AGO1 level declines; (B) more mAGO1 is targeted by let-7 RISC and directed to p-body for temporary storage, leading to a decrease in the (C) free form mAGO1 that are ready to be translated. The lower the oxygen availability, the quicker the change in mAGO1 takes place, the steeper the initial drop in total AGO1.

S2_Fig

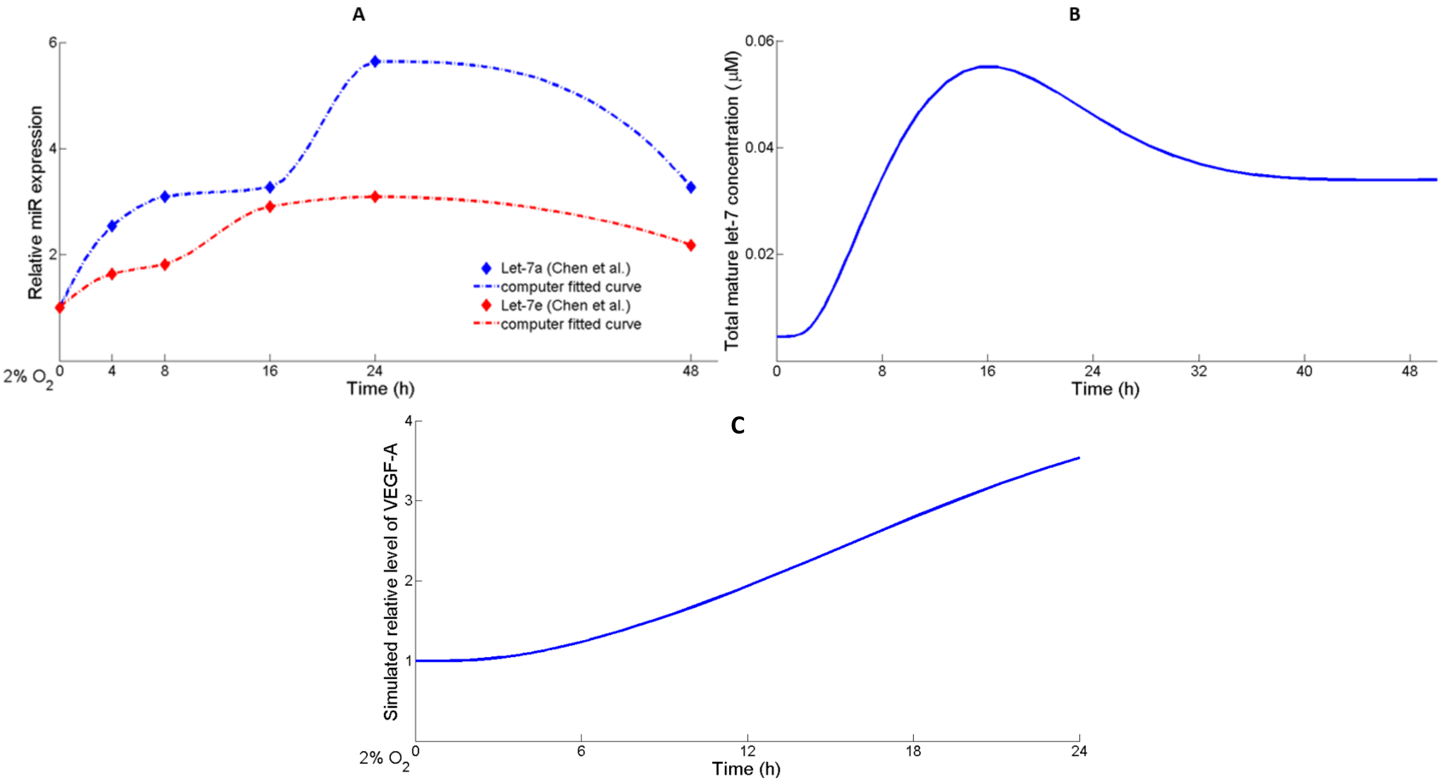

S2_Fig. Experimental time course profiles of let-7 and model simulations of intracellular let-7, VEGF in hypoxia. (A) Experimental quantification of total mature let-7s induced in 2% oxygen in endothelial cell by Chen et al. at discrete time points; values (symbols) are normalized to initial expression and dashed curves are computer interpolated trajectories [5]. (B) In agreement with the trend shown in the experimental data, model simulation predicts an initial steep rise and a subsequent drop in total let-7 level due in 2% oxygen. (C) Model simulation of intracellular VEGF predicts a 3.5 fold induction (relative to the normoxic baseline level) in response to a 24-hour exposure to 2% O_2.

S3_Fig

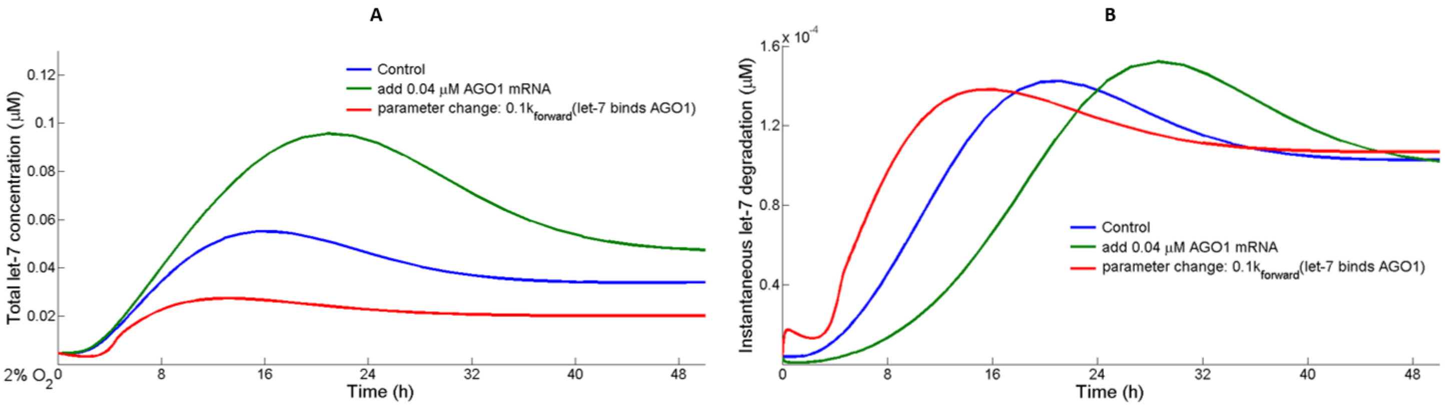

S3_Fig. AGO1 binds let-7 and prevents it from degradation. (A) Enforced overexpression of AGO1 binds more free form let-7 and stabilizes the total let-7 level in hypoxia, while a weak association between AGO1 and let-7 causes more let-7 degradation. (B) In the curves showing instantaneous degradation of let-7 in hypoxia, the case of weak AGO1/let-7 binding has larger let-7 degradation in the beginning; the case of AGO1 overexpression has much smaller let-7 degradation. Since the steady state levels of free let-7 are comparable in all three cases, the obvious differences in total let-7 levels are contributed by the additional let-7 stored in let-7/AGO1 complexes.

S4_Fig

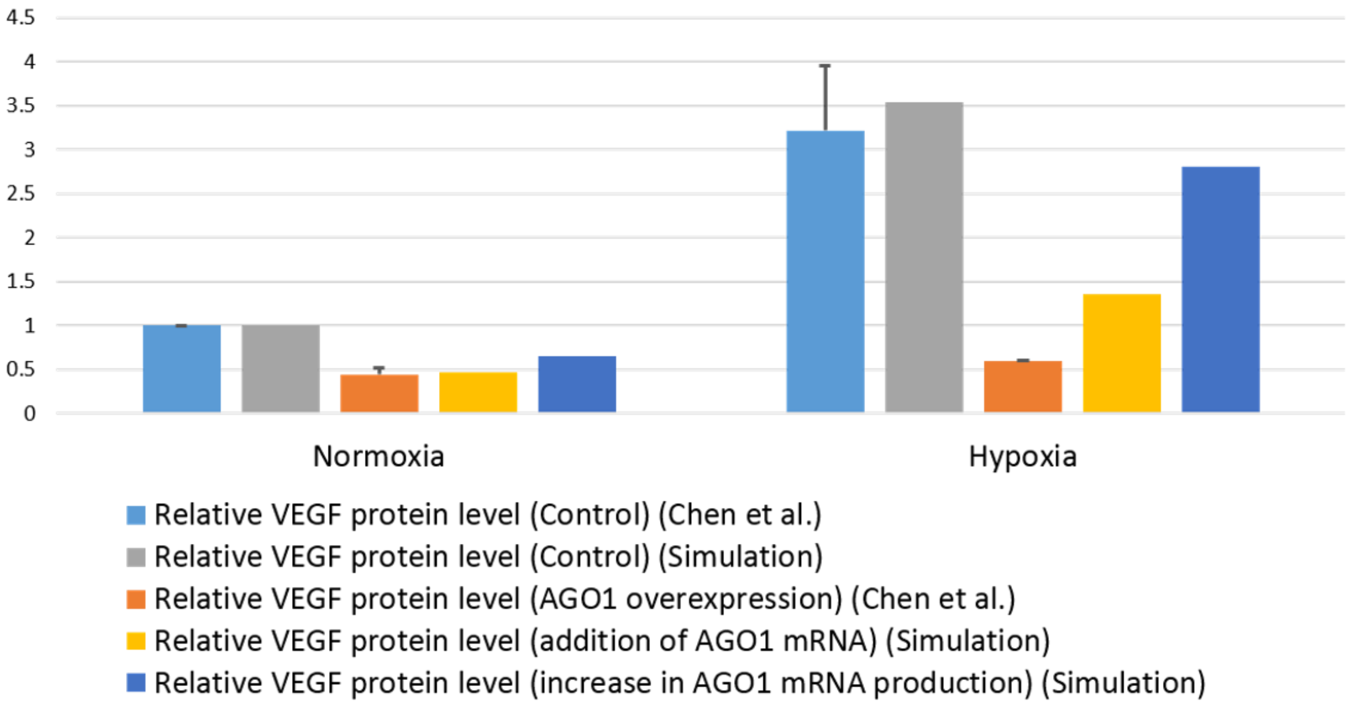

S4_Fig. AGO1 overexpression reduces VEGF production. Experiments by Chen et al. verified that AGO1 overexpression decreases VEGF protein level in HUVECs in normoxia and in hypoxia. We numerically quantified the Western blot data provided in literature [5]. Standard deviations were calculated and relative expressions were normalized to the normoxic VEGF protein level with no additional AGO1 introduced. Model simulations of AGO1 overexpression by two different approaches, increase the initial AGO1 mRNA by 0.08 µM or increase the AGO1 mRNA production rate by 50 fold, result in reductions of intracellular VEGF protein levels that are in qualitative agreement with the experiments. Because of the lack of relevant information in the experimental protocol, we can only approximate the values of the simulation parameters that were used to mimic the effect of *in vitro* AGO1 overexpression, and simulated VEGF protein levels at 24 hours were measured and compared to experimental Western blot quantifications.

S5_Fig

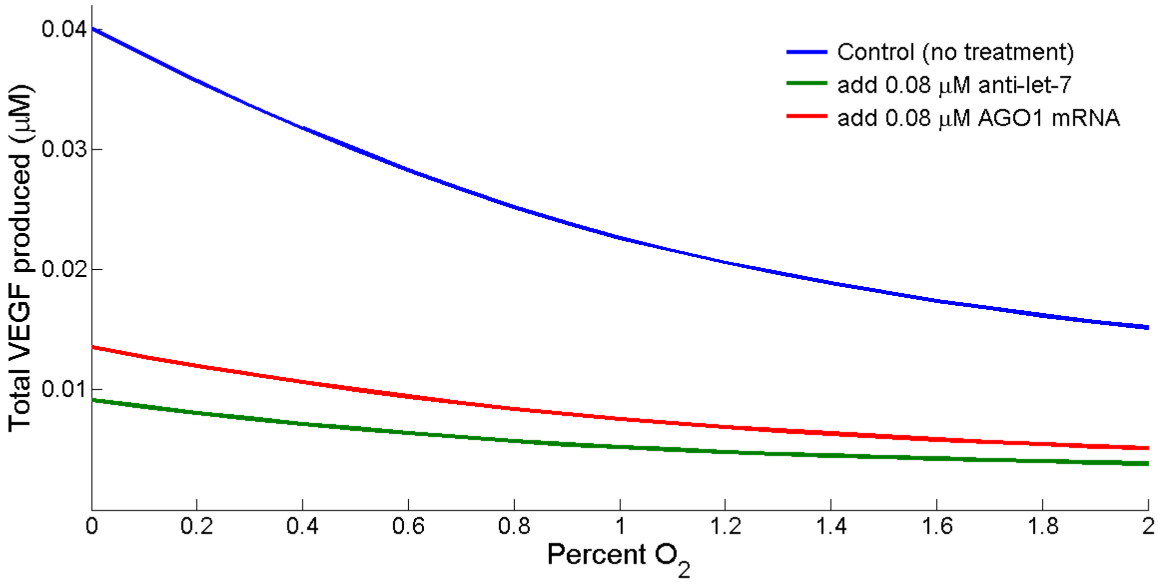

S5_Fig. Effect of anti-angiogenic strategies in extreme hypoxia. In extremely low O_2 concentrations (0-2%) that mimic tumor microenvironment, simulations show that using let-7 antagonists (green) or overexpressing AGO1 (red) significantly reduces the total VEGF synthesized during a 24-hour span by nearly four fold, compared to the untreated situation (blue).

S6_Fig

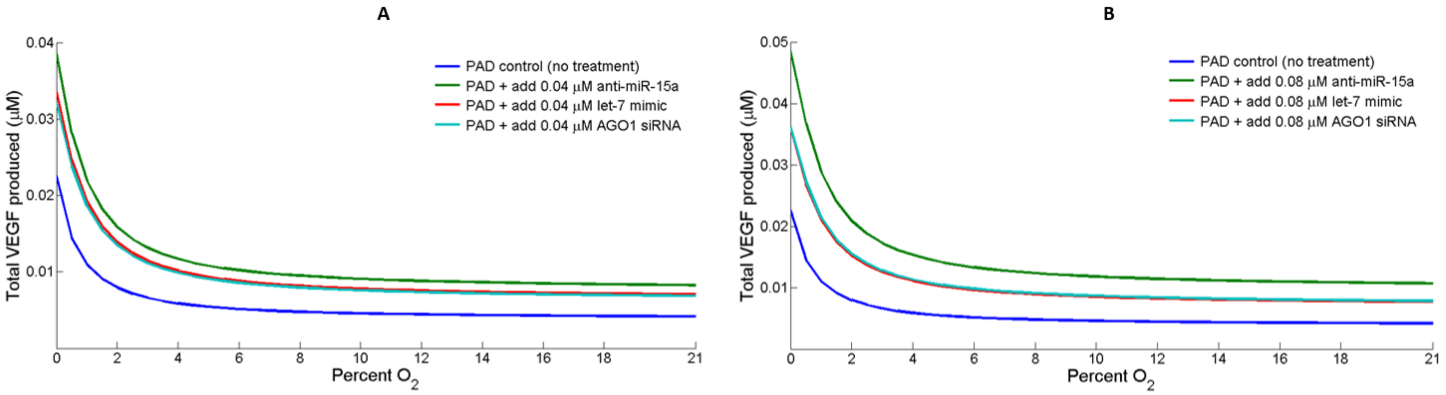

S6_Fig. Comparison between different treatments for PAD *in silico*. (A) Measurement of total VEGF produced in a 24-hour span in response to 0.04 μM of each treatment, and (B) 0.08 μM of each treatment. Since antagonizing miR-15a immediately releases VEGF mRNA from the miR-15a RISC while the other two strategies exert their effect indirectly, miR-15a antagonist is more effective in stimulating VEGF synthesis compared to let-7 mimic or AGO1 siRNA, at the same dose level in a simulated PAD environment.

S7_Fig

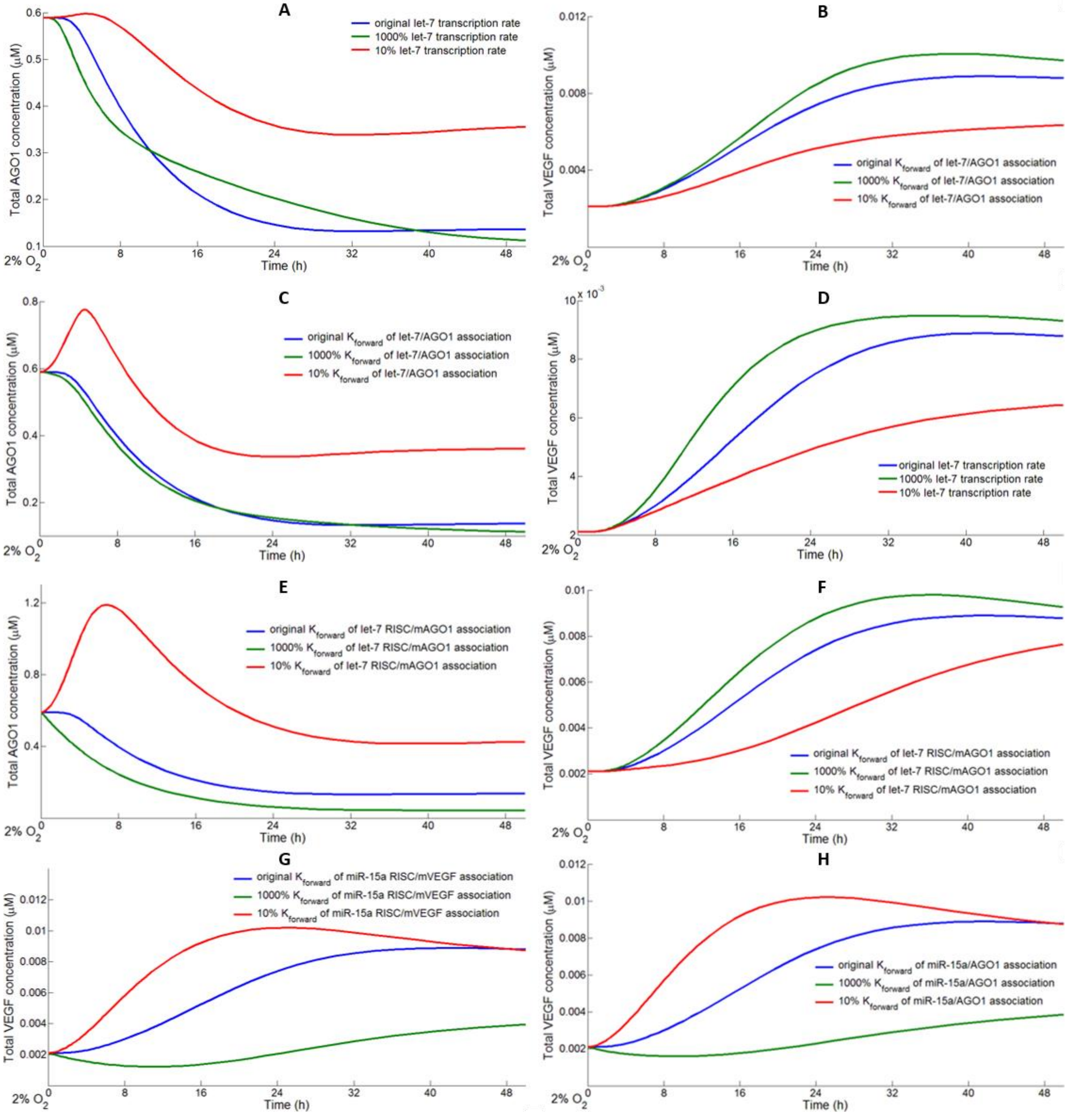
A
original let-7 transcription rate
1000% let-7 transcription rate
10% let-7 transcription rate
Total AGO1 concentration (μM)
Time (h)
2% O2
B
original Kforward of let-7/AGO1 association
1000% Kforward of let-7/AGO1 association
10% Kforward of let-7/AGO1 association
Total VEGF concentration (μM)
C
original Kforward of let-7/AGO1 association
1000% Kforward of let-7/AGO1 association
10% Kforward of let-7/AGO1 association
D
x 10-3
original let-7 transcription rate
1000% let-7 transcription rate
10% let-7 transcription rate
E
original Kforward of let-7 RISC/mAGO1 association
1000% Kforward of let-7 RISC/mAGO1 association
10% Kforward of let-7 RISC/mAGO1 association
F
original Kforward of let-7 RISC/mAGO1 association
1000% Kforward of let-7 RISC/mAGO1 association
10% Kforward of let-7 RISC/mAGO1 association
G
original Kforward of miR-15a RISC/mVEGF association
1000% Kforward of miR-15a RISC/mVEGF association
10% Kforward of miR-15a RISC/mVEGF association
H
original Kforward of miR-15a/AGO1 association
1000% Kforward of miR-15a/AGO1 association
10% Kforward of miR-15a/AGO1 association

S7_Fig. Additional sensitivity analysis of selected reaction rates affiliated with major species in the pathway. Modular sensitivity analysis presented in the article identified several reaction parameters that can most significantly influence the behaviors of the model, so here we performed additional analysis specific to these parameters. Varying the rate of let-7 transcription by $\underline{+}$10 fold result in different magnitudes of (A) AGO1 downregulation and (B) VEGF induction. Disrupting the association between let-7 and AGO1, rather than enforcing the binding, has a more profound impact on (C) AGO1 dynamics and (D) leads to a siginificant temporal delay in the induction of VEGF, while decreasing the forward binding rate of let-7 RISC with AGO1 mRNA causes a more significant change to (E) AGO1 profile and it further delayes (F) VEGF synthesis. Lastly, either varying the rate of VEGF targeting by miR-15a RISC or the rate of miR-15a/AGO1 association has very little impact on the time course AGO1 dynamics (data not shown), and both parameters, when varied by $\underline{+}$ 10 fold respectively, result in comparable VEGF expression curves (G-H). The initial rise in AGO1 dynamics observed in (C and E) is due to the fact that all three simulations are based on the same initial conditions, which allows more straightforward comparisons; still, the continuing decrease in AGO1 expression is the dominant behavior during the simulation span. These results reflect the intrinsic robustness of the model, which is demonstrated by these analysis to exhibit highly consistent dynamics/patterns with only minor quantitative changes upon parameter variations. Therefore, the major conclusions drawn based on the overall qualitative behavior of the key species in the model (e.g. AGO1, VEGF), in spite of the fact uncertainty exists in the parameter space for a reasonable range, would potentially remain unchanged.

S8_Fig

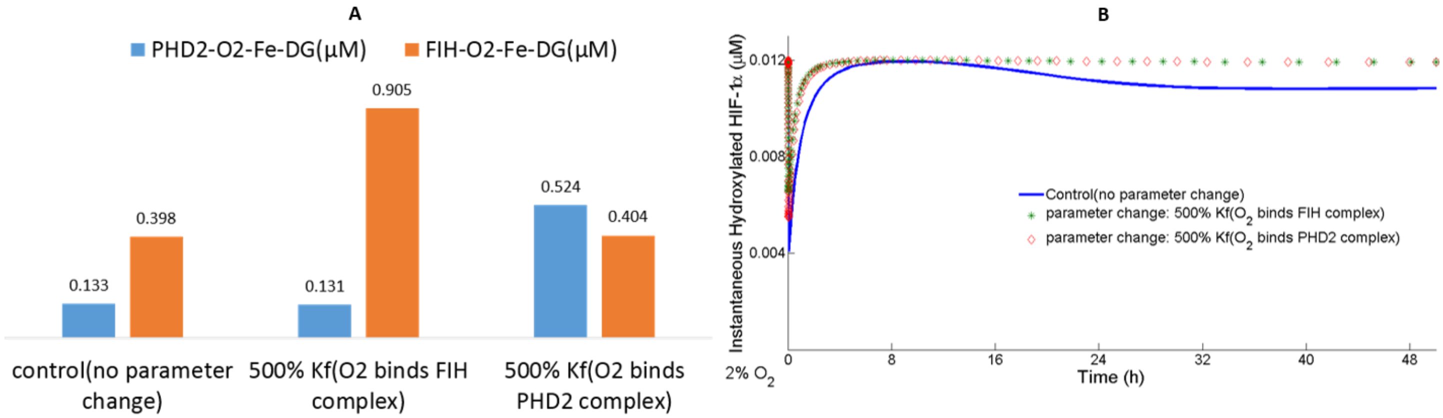

S8_Fig. Increased binding of O_2 with HIF hydroxylases reduces the effect of HIF stabilization in both normoxia and hypoxia. Here we selectively increase the forward binding rate of O_2 with hydroxylase 5 fold; in response to this, (A) in hypoxia (2% oxygen), steady state total compound formation of O_2 with hydroxylases increases, which (B) speeds up the instantaneous hydroxylation of HIF-1α during the simulation compared to the control case (blue curve). The hydroxylation rate indicated by green asterisks (increased FIH binding) does not differ significantly from the rate indicated by red diamonds (increased PHD2 binding), but these two cases have different HIF-1α initial conditions so the relative fold changes computed are not identical.

S9_Fig

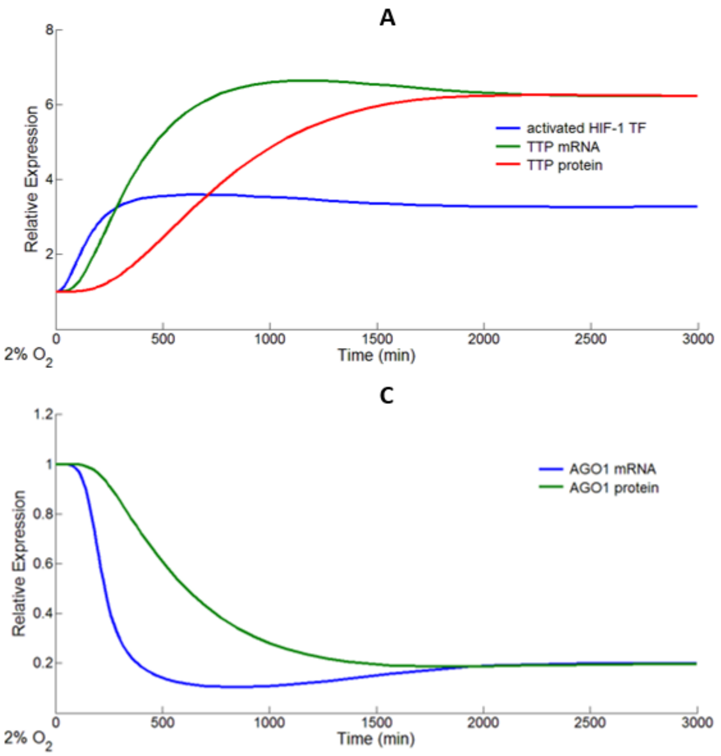

S9_Fig. Inherited time delays in the model and comparisons between ODE and DDE implementations. The computational model implemented in ordinary differential equations (ODEs) has inherited time delays in processes such as transcription and translation of (A) TTP, (B) VEGF, and (C) AGO1. (D) Re-calculating a subpart of the model, in this case the synthesis of TTP, by delayed differential equations (DDE) while increasing TTP tranlsation (v17) and TTP protein degradation (v16) rate by 4.5-fold produces time curves that are simliar to results obtained in the original ODE implementation. The faster production/degradation rates tested in the DDE approach give rise to a small difference in the TTP protein curve (an early overshoot), but its influence on the original model behavior is insignificant since the TTP protein steady states, which determine the final HIF-1α levels, are very similar in both approaches. This suggests that our model, an ODE system with tuned reaction parameters, can reproduce the effect of time delays in real biological systems, and that its core behaviors would not alter qualitatively if we implement the model in DDE with a new set of fitted parameters.

Reference

1. Kim TW, Yim S, Choi BJ, Jang Y, Lee JJ, Sohn BH, et al. Tristetraprolin regulates the stability of HIF-1alpha mRNA during prolonged hypoxia. Biochemical and biophysical research communications. 2010;391(1):963-8. doi: 10.1016/j.bbrc.2009.11.174. PubMed PMID: 19962963.
2. Ke Q, Costa M. Hypoxia-inducible factor-1 (HIF-1). Molecular pharmacology. 2006;70(5):1469-80. doi: 10.1124/mol.106.027029. PubMed PMID: 16887934.
3. Lando D, Peet DJ, Gorman JJ, Whelan DA, Whitelaw ML, Bruick RK. FIH-1 is an asparaginyl hydroxylase enzyme that regulates the transcriptional activity of hypoxia-inducible factor. Genes & development. 2002;16(12):1466-71. doi: 10.1101/gad.991402. PubMed PMID: 12080085; PubMed Central PMCID: PMC186346.
4. Forsythe JA, Jiang BH, Iyer NV, Agani F, Leung SW, Koos RD, et al. Activation of vascular endothelial growth factor gene transcription by hypoxia-inducible factor 1. Molecular and cellular biology. 1996;16(9):4604-13. PubMed PMID: 8756616; PubMed Central PMCID: PMC231459.
5. Chen Z, Lai TC, Jan YH, Lin FM, Wang WC, Xiao H, et al. Hypoxia-responsive miRNAs target argonaute 1 to promote angiogenesis. The Journal of clinical investigation. 2013;123(3):1057-67. doi: 10.1172/JCI65344. PubMed PMID: 23426184; PubMed Central PMCID: PMC3582133.
6. Ross CR, Brennan-Laun SE, Wilson GM. Tristetraprolin: roles in cancer and senescence. Ageing research reviews. 2012;11(4):473-84. doi: 10.1016/j.arr.2012.02.005. PubMed PMID: 22387927; PubMed Central PMCID: PMC3376680.
7. Bartel DP. MicroRNAs: genomics, biogenesis, mechanism, and function. Cell. 2004;116(2):281-97. PubMed PMID: 14744438.
8. Hebert SS, Papadopoulou AS, Smith P, Galas MC, Planel E, Silahtaroglu AN, et al. Genetic ablation of Dicer in adult forebrain neurons results in abnormal tau hyperphosphorylation and neurodegeneration. Human molecular genetics. 2010;19(20):3959-69. doi: 10.1093/hmg/ddq311. PubMed PMID: 20660113.
9. Yin KJ, Olsen K, Hamblin M, Zhang J, Schwendeman SP, Chen YE. Vascular endothelial cell-specific microRNA-15a inhibits angiogenesis in hindlimb ischemia. The Journal of biological chemistry. 2012;287(32):27055-64. doi: 10.1074/jbc.M112.364414. PubMed PMID: 22692216; PubMed Central PMCID: PMC3411046.
10. Eulalio A, Behm-Ansmant I, Schweizer D, Izaurralde E. P-body formation is a consequence, not the cause, of RNA-mediated gene silencing. Molecular and cellular biology. 2007;27(11):3970-81. doi: 10.1128/MCB.00128-07. PubMed PMID: 17403906; PubMed Central PMCID: PMC1900022.
11. Parker R, Sheth U. P bodies and the control of mRNA translation and degradation. Molecular cell. 2007;25(5):635-46. doi: 10.1016/j.molcel.2007.02.011. PubMed PMID: 17349952.
12. Zisoulis DG, Kai ZS, Chang RK, Pasquinelli AE. Autoregulation of microRNA biogenesis by let-7 and Argonaute. Nature. 2012;486(7404):541-4. doi: 10.1038/nature11134. PubMed PMID: 22722835; PubMed Central PMCID: PMC3387326.
13. Tokumaru S, Suzuki M, Yamada H, Nagino M, Takahashi T. let-7 regulates Dicer expression and constitutes a negative feedback loop. Carcinogenesis. 2008;29(11):2073-7. doi: 10.1093/carcin/bgn187. PubMed PMID: 18700235.
14. Stenvang J, Petri A, Lindow M, Obad S, Kauppinen S. Inhibition of microRNA function by antimiR oligonucleotides. Silence. 2012;3(1):1. doi: 10.1186/1758-907X-3-1. PubMed PMID: 22230293; PubMed Central PMCID: PMC3306207.
15. Qutub AA, Popel AS. A computational model of intracellular oxygen sensing by hypoxia-inducible factor HIF1 alpha. Journal of cell science. 2006;119(Pt 16):3467-80. doi: 10.1242/jcs.03087. PubMed PMID: 16899821; PubMed Central PMCID: PMC2129128.
16. Bissels U, Wild S, Tomiuk S, Holste A, Hafner M, Tuschl T, et al. Absolute quantification of microRNAs by using a universal reference. Rna. 2009;15(12):2375-84. doi: 10.1261/rna.1754109. PubMed PMID: 19861428; PubMed Central PMCID: PMC2779673.
17. Schwanhausser B, Busse D, Li N, Dittmar G, Schuchhardt J, Wolf J, et al. Corrigendum: Global quantification of mammalian gene expression control. Nature. 2013;495(7439):126-7. doi: 10.1038/nature11848. PubMed PMID: 23407496.

18. Yang E, van Nimwegen E, Zavolan M, Rajewsky N, Schroeder M, Magnasco M, et al. Decay rates of human mRNAs: correlation with functional characteristics and sequence attributes. Genome research. 2003;13(8):1863-72. doi: 10.1101/gr.1272403. PubMed PMID: 12902380; PubMed Central PMCID: PMC403777.
19. Gantier MP, McCoy CE, Rusinova I, Saulep D, Wang D, Xu D, et al. Analysis of microRNA turnover in mammalian cells following Dicer1 ablation. Nucleic acids research. 2011;39(13):5692-703. doi: 10.1093/nar/gkr148. PubMed PMID: 21447562; PubMed Central PMCID: PMC3141258.
20. Tuckerman JR, Zhao Y, Hewitson KS, Tian YM, Pugh CW, Ratcliffe PJ, et al. Determination and comparison of specific activity of the HIF-prolyl hydroxylases. FEBS letters. 2004;576(1-2):145-50. doi: 10.1016/j.febslet.2004.09.005. PubMed PMID: 15474027.
21. Liu Q, Xu Z, Mao S, Chen W, Zeng R, Zhou S, et al. Effect of hypoxia on hypoxia inducible factor-1alpha, insulin-like growth factor I and vascular endothelial growth factor expression in hepatocellular carcinoma HepG2 cells. Oncology letters. 2015;9(3):1142-8. doi: 10.3892/ol.2015.2879. PubMed PMID: 25663870; PubMed Central PMCID: PMC4315007.
